# Biochemical Pathways Triggered by Antipsychotics in Human Oligodendrocytes: Potential of Discovering New Treatment Targets

**DOI:** 10.3389/fphar.2019.00186

**Published:** 2019-03-05

**Authors:** Caroline Brandão-Teles, Valéria de Almeida, Juliana S. Cassoli, Daniel Martins-de-Souza

**Affiliations:** ^1^Laboratory of Neuroproteomics, Department of Biochemistry and Tissue Biology, Institute of Biology, University of Campinas, Campinas, Brazil; ^2^Faculdade de Palmas, Palmas, Brazil; ^3^UNICAMP’s Neurobiology Center, Campinas, Brazil; ^4^Instituto Nacional de Biomarcadores em Neuropsiquiatria, Conselho Nacional de Desenvolvimento Científico e Tecnológico, São Paulo, Brazil

**Keywords:** schizophrenia, mechanism of action, proteomics, biomarkers, chlorpromazine, haloperidol, quetiapine, risperidone

## Abstract

Schizophrenia is a psychiatric disorder that affects more than 21 million people worldwide. It is an incurable disorder and the primary means of managing symptoms is through administration of pharmacological treatments, which consist heavily of antipsychotics. First-generation antipsychotics have the properties of D_2_ receptor antagonists. Second-generation antipsychotics are antagonists of both D_2_ and 5HT_2_ receptors. Recently, there has been increasing interest in the effects of antipsychotics beyond their neuronal targets and oligodendrocytes are one of the main candidates. Thus, our aim was to evaluate the molecular effects of typical and atypical drugs across the proteome of the human oligodendrocyte cell line, MO3.13. For this, we performed a mass spectrometry-based, bottom-up shotgun proteomic analysis to identify differences triggered by typical (chlorpromazine and haloperidol) and atypical (quetiapine and risperidone) antipsychotics. Proteins which showed changes in their expression levels were analyzed *in silico* using Ingenuity^®^ Pathway Analysis, which implicated dysregulation of canonical pathways for each treatment. Our results shed light on the biochemical pathways involved in the mechanisms of action of these drugs, which may guide the identification of novel biomarkers and the development of new and improved treatments.

## Introduction

Schizophrenia is a chronic and debilitating psychiatric disorder characterized by positive (e.g., hallucinations and delusions), negative (e.g., anhedonia, alogia, apathy, and poor self-care), and cognitive (e.g., deficits in executive function, working memory, and recognition memory) symptoms. The risk for schizophrenia is 0.7% in the broad population but increases with the degree of genetic relationship (Hayashi-Takagi, 2016). Treatment is based on administration of antipsychotics to reduce the recurrence and severity of psychosis and improve general symptoms, thereby providing some degree of improvement in quality of life for patients. Antipsychotics can be divided into two categories known as typical (first-generation) and atypical (second-generation) drugs. A common pharmacological property presented by these classes is the blockage of the dopamine D_2_ receptor (Shen, 1999; Leucht et al., 2009; Kantoff et al., 2010).

First-generation antipsychotics such as chlorpromazine and haloperidol are effective in reducing positive symptoms, but they can cause severe side effects such as extrapyramidal symptoms and tardive dyskinesia. Additionally, these drugs do not treat negative and cognitive symptoms, which contribute to most of the deficiency associated with schizophrenia (Lieberman et al., 2005). Second-generation antipsychotics like quetiapine and risperidone are also effective to attenuate positive symptoms and are more effective in reducing negative symptoms compared to first-generation drugs. However, severe side effects such as weight gain, metabolic syndrome, and sedation may occur (Leucht et al., 2009).

The pathophysiology of schizophrenia has been associated with disturbances in several neurotransmitter systems. One of the most well-established theories is the dopamine hypothesis, which agrees with current antipsychotics targeting dopaminergic systems. This and other hypotheses have normally implicated neurons, although other molecular mechanisms involving different cell types in the brain may be associated with the pathophysiology of schizophrenia (Coyle, 1996; Ju and Cui, 2015). Oligodendrocytes are the cells responsible for myelination in the central nervous system (CNS) through expression of genes that encode myelin structural proteins in a specific and regulated manner, and myelination of axon fibers by oligodendrocytes is essential for the rapid conduction of action potentials (Buntinx et al., 2003).

Scientific evidence suggests that one of the reasons for neuronal disconnection is due to disruptions in myelination. This could be due to poor functioning of oligodendrocytes. Such a disruption may lead to dysfunctions of perception, behavior and cognition as seen in schizophrenia as reviewed by Takahashi (Takahashi et al., 2011). It has already been shown that there is a prominent reduction in the density of oligodendrocytes in the prefrontal cortex of these patients (Hof et al., 2003; Uranova et al., 2004). In addition, it has already been seen in the dorsolateral prefrontal cortex alteration in expression levels in genes related to myelination of patients with schizophrenia compared to control (Hakak et al., 2001). Evidence for myelin-related changes in schizophrenia is also provided through studies with animal models. Animals treated with NMDA receptor antagonists (NMDAr), a pharmacological model of schizophrenia (Paulson et al., 2003; Neill et al., 2010) showed a decrease in the total volume of white matter and corpus callosum compared to control animals. Furthermore, levels of myelin basic protein (MBP) in these animals were found to be decreased (Xiu et al., 2014). In addition, transcriptomic studies using post mortem brain tissue have implicated that genetic variation in OLIG2, CNP, MAG, and MOG can be associated to myelination and oligodendrocyte function in schizophrenia (Hakak et al., 2001; Georgieva et al., 2006). It is important to mention that myelination continues throughout development of the young adult brain, coinciding with the average age of onset of the pathology. We have demonstrated recently an increase in the levels of many proteins in cultured oligodendrocytes treated with MK-801, an antagonist of the NMDA receptor, suggesting that these cells may be targets for antipsychotics (Cassoli et al., 2016).

Here we performed a quantitative proteomic analysis to investigate changes in protein expression triggered by antipsychotics in a cell line of human oligodendrocytes (MO3.13). The effects of two first-generation (chlorpromazine and haloperidol) and two second-generation (quetiapine and risperidone) antipsychotics were investigated. Our aim was to better understand the biochemical pathways involved in the mechanisms of action of these drugs in attempt to find new biomarkers and targets which may increase our understanding of the disease and therapeutic response and assist in the development of more specific and improved treatments for patients with schizophrenia.

## Experimental Procedures

### Cell Culture, Treatments, and Proteome Extraction

Human hybrid MO3.13 cells are classified as an immature oligodendrocyte cell line (Buntinx et al., 2003; Cassoli et al., 2017). Our group using a 2D liquid chromatographic strategy combined with UDMS^E^ acquisitions have established a complete dataset of proteins expressed by the MO3.13 cells. More than 10,000 proteins expressed in this cell line have been identified, and it is possible to find the receptors mentioned in this study, such as the dopamine D_2_ receptor, target of antipsychotics (Cassoli et al., 2017).

MO3.13 cells were grown in DMEM medium supplemented with 0.5% penicillin/streptomycin (Sigma-Aldrich, St. Louis, MO, United States) and 10% heat-inactivated fetal bovine serum (Life Technologies, Darmstadt, Germany) at 37°C in a humidified atmosphere containing 5% CO_2_ as described previously (Brandão-Teles et al., 2017). The cells were treated with each antipsychotic once and collected after 8h as follows with respect to dosage and drug: Group 1–10 μM chlorpromazine; Group 2–50 μM haloperidol; Group 3–50 μM quetiapine; Group 4–50 μM risperidone; Group 5–chlorpromazine and haloperidol vehicle solution (0.01 M HCl); Group 6 – quetiapine and risperidone vehicle solution (DMSO). Each treatment was done in biological triplicate. The antipsychotic doses were chosen as described previously (Martins-de-Souza et al., 2011). MO3.13 cells were centrifuged at 1,200 *g* for 5 min and the pellets homogenized in a lysis buffer consisting of 6 M urea, 2 M thiourea, 10 mM DTT, with protease and phosphatase inhibitors, 0.1 mM sodium pervanadate (lysis buffer). Protein lysates were centrifuged at 14,000 *g* for 45 min at 4°C in order to remove pelleted lipids and other vestiges. The supernatants were collected, desalted and concentrated as described in Brandão-Teles et al. (2017). Protein concentrations were determined by Qubit^®^ Protein Assay Kit.

### NanoLC-ESI MS/MS

Proteomic analyses were performed in a bidimensional microUPLC tandem nanoESI-UDMS^E^ platform by multiplexed data-independent acquisitions experiments, using a 2D-RP/RP Acquity UPLC M-Class System (Waters Corporation, Milford, MA, United States) coupled to a Synapt G2-Si mass spectrometer (Waters Corporation, Milford, MA, United States). The samples were fractionated using a one-dimension reversed-phase approach. Peptide samples (0.5 μg) were loaded into a M-Class HSS T3 column (100 Å, 1.8 μm, 75 μm × 150 mm, Waters Corporation, Milford, MA, United States). The fractionation was achieved using an acetonitrile gradient from 7 to 40% (v/v) over 95 min at a flow rate of 0.4 μL/min directly into Synapt G2-Si mass spectrometer. For every measurement, MS and MS/MS data were acquired in positive resolution mode with a resolving power around 25,000 FWHM. Ion mobility separation of precursor ions method (Geromanos et al., 2012) was used over a range of 50–2000 m/z and a cross-section resolving power of at least 40 Ω/ΔΩ. Precursor ion information was collected in low-energy MS mode by applying a constant collision energy of 4 eV in the range of 50–2000 m/z. Fragment ion information was obtained in the elevated energy scan using drift-time specific collision energies as detailed previously (Cassoli et al., 2017). The spectral acquisition time in each mode was 0.6 s with a 0.05 s-interscan delay, resulting in an overall cycle time of 1.3 s for the acquisition of one cycle of low and high energy data. The lock mass channel was sampled every 30 s. The mass spectrometer was calibrated using a human [Glu1]-Fibrinopeptide B (785.8426 m/z) solution delivered through the reference sprayer of the NanoLock Spray source. All proteomics analyses were run in technical duplicate.

### Data Processing and Database Searches

Proteins were identified and quantified using dedicated algorithms and searching against the UniProt Human Proteomic Database of *Homo sapiens*, version 2018/02 (Li et al., 2009; Cassoli and Martins-de-Souza, 2017). The databases used were reversed “on the fly” during the database queries by the software to assess the false-positive identification rate. For correct spectral processing and database searching conditions, we used the Progenesis QI for Proteomics software package with Apex3D, Peptide 3D, and Ion Accounting informatics (Waters Corporation). This software starts with loading of the LC-MS data, followed by alignment and peak detection, which creates a list of interesting peptide ions that are explored within Peptide Ion Stats by multivariate statistical methods. The processing parameters used were 150 counts for the low-energy threshold, 50.0 counts for the elevated energy threshold, and 750 counts for the intensity threshold. Automatic alignment of the runs (all runs in the experiment was assessed for suitability) was used for the processing. In peak picking, it was used 5 as maximum ion charge and the sensitivity value was selected as 4. Moreover, the following parameters were considered in identification of peptides/proteins: (1) digestion by trypsin with at most one missed cleavage; (2) variable modification by oxidation (M) and fixed modification by carbamidomethyl (C); and (3) a false discovery rate (FDR) less than 1%. Two or more ion fragments per peptide, five or more fragments per protein and one or more peptides per protein were required for ion matching. Data were analyzed by on-way analysis of variance (ANOVA) with treatment factor *p*-value <0.05 compared to vehicle group. Identifications that did not satisfy these criteria were rejected. The experiment design was defined (Group 1–6) and label free protein quantitation was done using Hi-N (*N* = 3) method.

### Analysis *in silico*

For interpreting the functional significance of differentially expressed proteins, their UniProt accession IDs were uploaded into the Ingenuity Pathways Knowledgebase (IPKB) through the algorithm Ingenuity Pathway Analysis (IPA, Ingenuity Systems, Qiagen, Redwood, CA, United States^1^) to determine potential interactions between these proteins, between these proteins and other proteins, to canonical pathways and to disease lists contained in the IPKB. The parameters used were the software default and *p*-value less than 0.05. Additional information can be found on Ingenuity Systems’ website^1^.

### Immunohistochemistry

MO3.13 cells for immunohistochemistry (adapted from Taraboletti et al., 2017) were grown in circular glass microscope slides in a six-well plate at a density of 3,5 × 10^4^ and treated as described previously. After the treatments, the cells were initially fixed with 4% paraformaldehyde for 20 min, washed three times and conserved in PBS. The microscope slides were washed in PBS three times and were then incubated with glycine 0.1 M for 30 min. After three additional washes, the cells were blocked for 5 min in PBS containing 10% fetal bovine serum. The cells were then incubated at 4°C overnight with primary antibodies in blocking solution (Taraboletti et al., 2017). We used the following primary antibodies: Anti-Cytochrome C antibody (1:400; Abcam; ab13575); and mTOR (1:400; Cell Signaling; 2983S). The cells were then washed with PBS and incubated with secondary antibodies and DAPI (1:100) for 1h at 37°C. The secondary antibodies were Alexa Fluor 594 goat anti-mouse or anti-rabbit used at a dilution of 1:400. The cells were fixed using mounting medium (Dako Faramount Aqueous Mounting Medium, Ref S3025) and imaged using an Cytation 5, BioTek and quantified using Fiji: an open-source platform for biological-image analysis. GraphPad Prism software version 8.0 was used to perform the statistical analyses.

### Oxidative Stress Indicator

MO3.13 cells were seeded on 12-well plate at a density of 4,5 × 10^4^ per well. After 48 h the cells were treated with Haloperidol for 4 h as described previously. After the treatment we added CM-H2DCFDA, according to the manual description (1:1000; Thermo; C6827), for 10 min in the dark to analyze the reactive oxygen species. The wells were, then, washed three times with PBS and 0.5 ml of DMEM supplemented with 0.5% penicillin/streptomycin and 10% heat-inactivated fetal bovine serum was added to each well. Fluorescence was measured using a Cytation 5 (BioTek).

## Results

**FIGURE 1:**
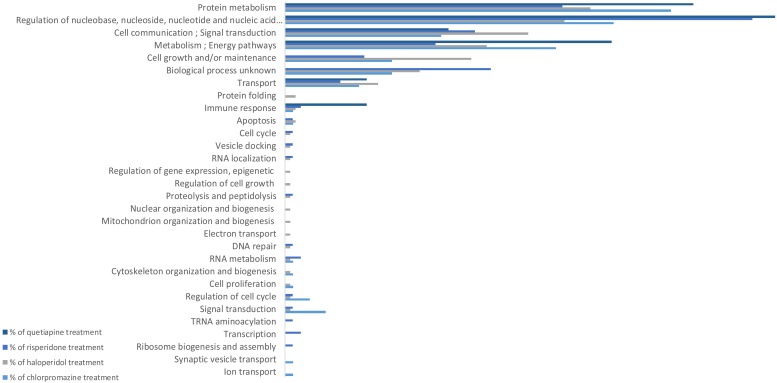
GO biological processes affected by antipsychotic treatment in MO3.13 cell cultures.

All antipsychotics affected the expression levels of some proteins (Figure 1) and consequently triggered changes in several biological processes, according to the *in silico* IPA profiling. Some of these differences were common among treatments and others were specific to each antipsychotic analyzed.

We identified in chlorpromazine treatment a total of 1138 proteins, of these 195 proteins presented changes in the abundance. In the case of haloperidol, we identified 1252 proteins with 316 presented different levels, compared to the levels of these proteins in untreated control cells (Supplementary Tables S1, S2, respectively). Proteins with different abundances affected 77 and 105 canonical pathways in cells treated with chlorpromazine and haloperidol, respectively (Supplementary Table S3). For atypical antipsychotics, in the quetiapine treatment we identified a total of 2201 proteins in which 19 proteins have their expression altered, while risperidone we identified 1705 proteins, of these 197 proteins presented changes in the abundance (Supplementary Tables S4, S5, respectively). These proteome changes were implicated in 17 and 32 conical pathways, respectively (Supplementary Table S6).

Figure 1 shows the biological processes which were affected both uniquely and in common by the tested antipsychotics. All antipsychotics caused changes in protein metabolism. The quetiapine treatment affected fewer biological processes compared to other antipsychotics, reflected by the lowest number of proteins expressed at different levels in the MO3.13 cells. Most of the differentially expressed proteins in quetiapine treatment are associated with the regulation of nucleobase, nucleoside, nucleotide and nucleic acid metabolism. Some processes were affected by only one treatment, which are: ion transport and synaptic vesicle transport, by chlorpromazine; electron transport, mitochondrial organization and biogenesis, nuclear organization and biogenesis, regulation of cell growth, and regulation of gene expression (epigenetic), by haloperidol; and ribosome biogenesis and assembly, and transcription pathways by risperidone albeit at a lower scale.

Proteins identified with different abundances were analyzed at IPA according to their canonical pathways. We only considered pathways with *p*-value less than 0.05. For typical antipsychotics, haloperidol modulated more canonical pathways (105) than chlorpromazine treatment (77) (Supplementary Table S3). In addition, 23,8% of the pathways were altered by both drugs, suggesting similarities in their mechanisms of action.

For atypical antipsychotics, no overlaps in the canonical pathways affected were observed. Quetiapine only enriched 17 canonical pathways, while the risperidone treatment led to an enrichment of 32 canonical pathways. Compared to typical antipsychotics, 2.3 and 5.2% of the pathways enriched by chlorpromazine and haloperidol, respectively, overlapped with those triggered by risperidone, but no overlap was observed between risperidone and quetiapine. Although presenting specific pathway differences, this does not rule out the possibility that risperidone may eventually use routes similar to typical antipsychotics while modulating the MO3.13 cell response. Depending on the dosage, risperidone may be considered clinically as a typical antipsychotic and this could be due to the high specificity of the effects induced by the risperidone treatment.

**FIGURE 2:**
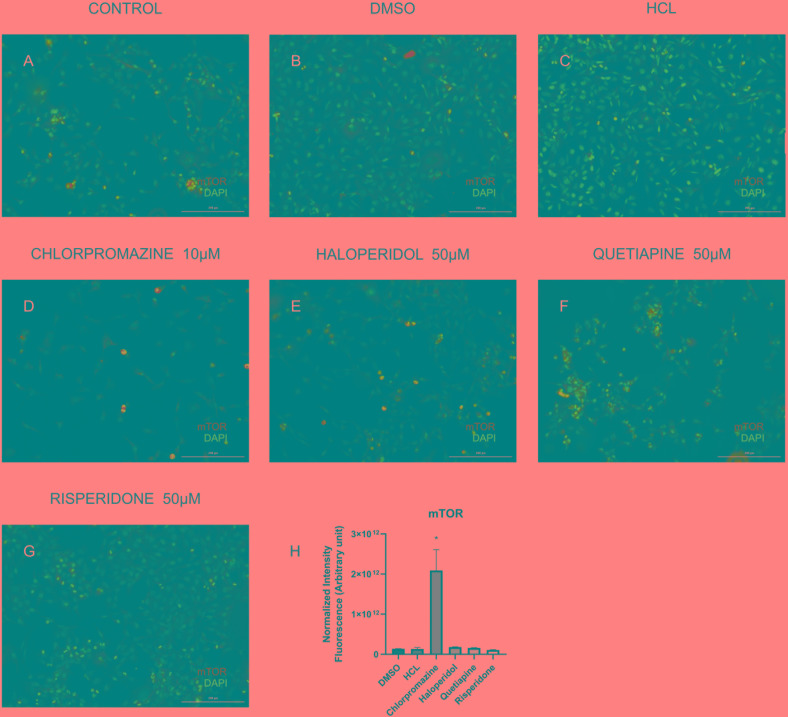
Representative images of MO3.13 cells treated for 8 h **(A)**, vehicles (DMSO, HCl; **B,C**), chlorpromazine 10 μM **(D)**, haloperidol 50 μM **(E)**, quetiapine 50 μM **(F)**, and risperidone 50 μM **(G)** stained for mTOR (green)/Dapi (blue). **(H)** Statistic chart showing normalized intensity fluorescence for mTOR for each treatment (^∗^*P* < 0.05). Scale bars = 200 μm.

**FIGURE 3:**
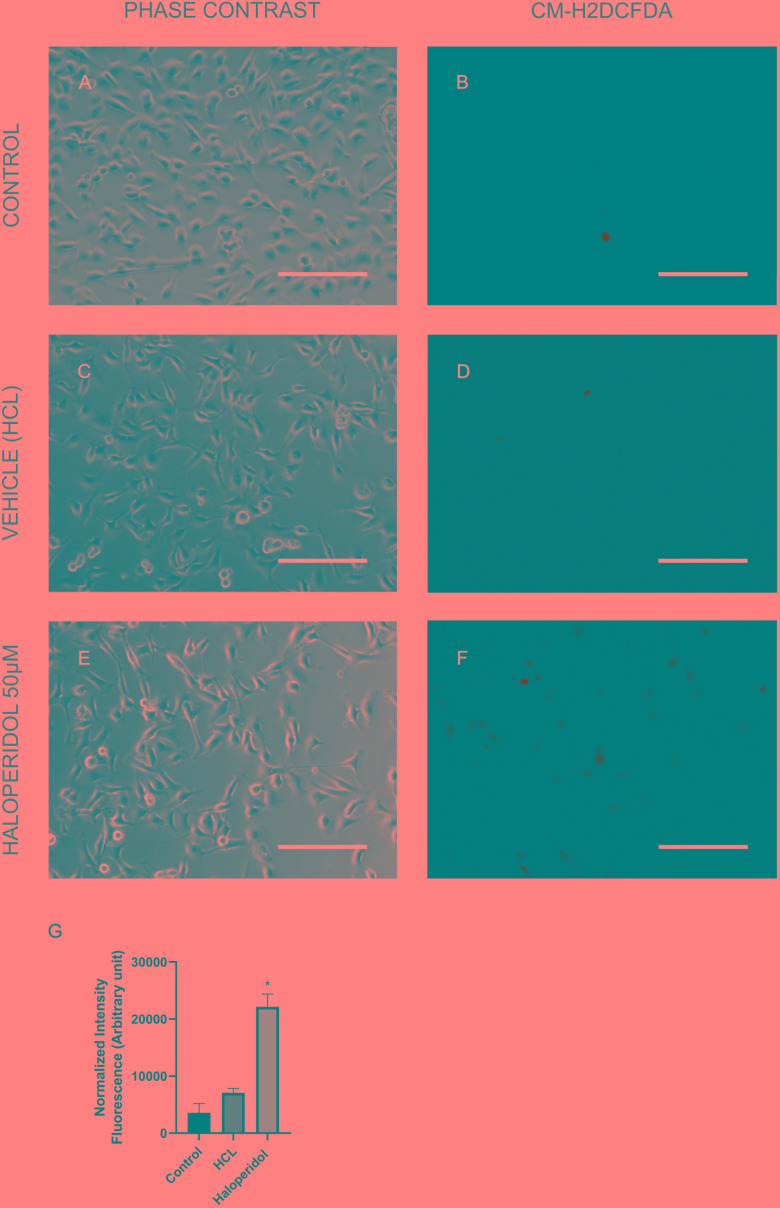
MO3.13 after treatment with control **(A,B)**, vehicle (HCL; **C,D**), and haloperidol 50 μM **(E,F)** for 4 h, and incubation with CM-H2DCFDA. Fluorescence is shown here in false colors and green for CM-H2DCFDA. **(G)** Statistic chart showing normalized intensity fluorescence for CM-H2DCFDA for each treatment (^∗^*P* < 0.05). Scale bars = 200 μm.

In order to validate some of the proteins we found differentially expressed in the treatments we performed immunocytochemistry for mTOR and cytochrome C. Our results showed that although chlorpromazine, haloperidol, and risperidone altered proteins belonging to mTOR pathways, only chlorpromazine affected mTOR protein directly (Figure 2). We found an increase in cytochrome C expression promoted by quetiapine treatment (Supplementary Figure S1). Additionally, to detect reactive oxygen species (ROS) in MO3.13 after the treatment with haloperidol we added CM-H2DCFDA, a cell permeable, non-fluorescent precursor of DCF, and read in the fluorescence microplate reader (Eruslanov and Kusmartsev, 2010). We observed that, compared to vehicle, haloperidol increased ROS production in the cells (Figure 3).

## Discussion

Most of what we know about antipsychotic mechanisms of action was discovered originally through effects on receptors in neuronal studies. However, fewer studies have been carried out investigating the effects of these drugs on glial cells. Considering the growing interest in the role of oligodendrocytes in neurotransmission, it makes sense to characterize the modulatory effects of antipsychotics on the biological processes of these cells. Thus, we investigated the acute response of healthy oligodendrocytes to both first- and second-generation antipsychotics through identification of proteins that were changed subsequently in their abundance. Here we demonstrated that antipsychotics affected in a different manner the proteome of oligodendrocyte. This information can be observed in IPA analyses through the canonical pathways’ ratio, that means the ratio of the number of proteins affected by treatments divided by the total number of proteins that belong to the same pathway. The canonical pathway coverage ratios for each of the treatment types ranged from 0.02–1 for haloperidol, 0.02–0.5 for chlorpromazine and risperidone, and 0.008–0.06 for quetiapine with ratios closer to 1 indicating higher coverage.

### Common Effects

#### Spliceosome

**FIGURE 4:**
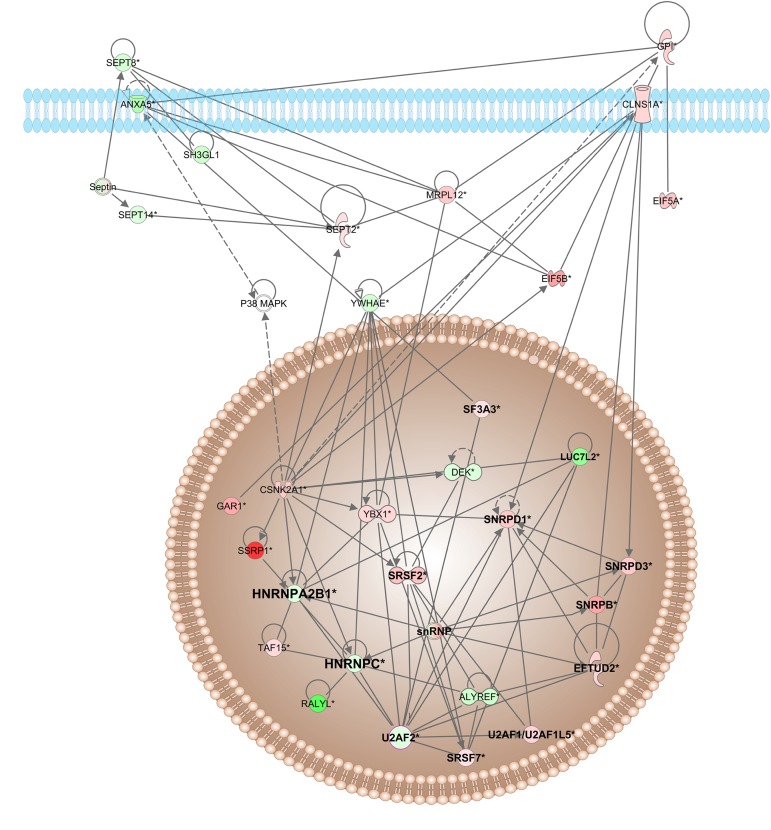
Network interactions, and their interactors, of differentially expressed proteins of haloperidol-treated oligodendrocytes. The network was generated from differentially expressed proteins by IPA. Colored interactors represent proteins previous found in the proteome. Highlighted proteins were belonging to splicing machinery. Full and dashed lines depict direct and indirect connections, respectively.

The spliceosome machinery is composed of five different ribonucleoprotein (RNP) subunits and numerous protein cofactors that together will participate in the splicing process, i.e., removal of the introns in the pre-mRNA (Matera and Wang, 2014). In this study, proteins belonging to spliceosome machinery were affected by all treatments. Among the proteins identified, chlorpromazine increased levels of proteins belonging to nuclear heterogeneous ribonucleoprotein family (hnRNP), such as hnRNPC, nhRNPK, hnRNPA1L2, and hnRNPC, and decreased levels of hnRNPA3. The haloperidol treatment also increased the levels of hnRNPF, hnRNPA3, and decreased hnRNPH2 (Figure 4). Most of the proteins affected by risperidone treatment were decreased, such as hnRNPH2, hnRNPH, hnRNPD, hnRNPA1, hnRNPU, hnRNPM, and hnRNPA0. Quetiapine treatment only altered the levels of two proteins, the Pre-mRNA-processing-splicing factor (PRPF8) and U6 snRNA-associated Sm-like protein (LSM2). Recently, we observed changes in eight proteins belonging to the hnRNP family in oligodendrocytes treated with clozapine (Cassoli et al., 2016). We also found altered levels of hnRNPC, hnRNPK and hnRNPU in *post mortem* samples of the anterior temporal lobe (ATL) from patients with schizophrenia (Saia-cereda and Santana, 2017). Additionally, we found changes in hnRNPC levels in the temporo-posterior gyrus of patients with schizophrenia (Martins-de-Souza et al., 2009). Finally, Iwata et al. (2011) reported that overexpression of the hnRNP C2 variant leads to a decrease in MBP expression. There are few studies linking spliceosome machinery mutations and psychiatric diseases, probably due to the critical role for survival (Glatt et al., 2011).

#### EIF2

Another effect of antipsychotics is related to the translation initiation factors, an important group of proteins required for protein synthesis on ribosomes. EIF2 (eukaryotic initiation factor-2) mediates the binding of Met-tRNA to the ribosome in a GTP-dependent manner (Carter, 2007). While chlorpromazine and haloperidol increased the expression most of proteins associated with EIF2 signaling, risperidone treatment decreased this protein. Although quetiapine treatment did not affect EIF2 signaling, this data suggests that first and second antipsychotics may have differential effects on protein synthesis.

#### mTOR

The mammalian target of rapamycin (mTOR) signaling pathway plays an important role in regulation of protein synthesis, mainly in neurodevelopment and synaptic plasticity (Yoon et al., 2008). One study observed that acute treatment with the NMDA receptor antagonist MK-801 resulted in increased phosphorylation of proteins in the mTOR-p70S6K pathway in rat frontal cortex (Yoon et al., 2008). Another investigation showed that haloperidol treatment appears to activate the AKT-mTORC1 pathway leading to changes in protein synthesis (Bowling et al., 2014). We observed that chlorpromazine, haloperidol and risperidone affected proteins belonging to mTOR pathway. Most proteins affected by chlorpromazine are increased, while treatment with risperidone decreased protein levels. In contrast, treatment with haloperidol similarly affected the proteins, both raising and decreasing their expression. Additionally, we could observed that only chlorpromazine affected directly the levels of mTOR (Figure 3). These differential effects could be due to variations in brain regions and cell types as we have specifically investigated an oligodendrocyte cell line in this study and because the treatments have many different mechanisms of action.

#### Ubiquitination Pathway

We also observed that the treatment with chlorpromazine and haloperidol resulted in alterations in the levels of proteins associated with the ubiquitination pathway. The treatment with chlorpromazine increased levels of heat shock family proteins (HSP90AA1, HSP90B1, HSPA6, and HSPE1) and proteins belonging to the proteasome (HSPE1, PSMC5, PSMD3, and PSMD13). The treatment with haloperidol also increased levels of proteins belonging to heat shock family (HSPA4, HSPA9, HSPA1A/HSPA1B, and HSPA4L) and proteasome proteins (PSMA6, PSMB6, PSMC2, PSMD4, PSMD7, and PSMD12). The main role of ubiquitination pathway is protein degradation through the conjugation of various portions of ubiquitin to the target protein followed by degradation of the protein bound to the polyubiquitin chain by the 26S proteasome complex (Ryan et al., 2006). Studies have shown decreased expression of genes related to this pathway in the dentate granule neurons and prefrontal cortex of schizophrenia patients (Middleton et al., 2002; Altar et al., 2005). Another study which carried out a blood-based microarray analysis found changes in the ubiquitin proteasome pathway of schizophrenia patients (Bousman et al., 2010). We suggest that chlorpromazine and haloperidol may restore the levels of the proteins involved in the ubiquitin pathway in schizophrenia, possibly aiding cellular processes regulated by this pathway such as signal transduction, synaptic plasticity, intracellular trafficking, endocytosis, DNA repair, and neural activity.

#### Energy Metabolism

Multiple studies have demonstrated a dysregulation in energy metabolism in schizophrenia. Proteomics analysis of *post mortem* brains of patients with schizophrenia showed alterations in the levels of energy metabolism-associated proteins, such as aldolase C (ALDOC), enolase 2 (ENO2), and glyceraldehyde-3-phosphate dehydrogenase (GAPDH) (Saia et al., 2015). Another investigation using samples of *post mortem* hippocampus of patients with schizophrenia found that glycolysis- and gluconeogenesis-associated proteins were altered, as seen by decreased levels of ALDOC and increased ENO1 (Schubert et al., 2015). Changes in energy metabolism have also been implicated in the effects of antipsychotics in oligodendrocytes. A study showed that haloperidol increased the amount of glucose and decreased the lactate levels present in the extracellular medium of cultured oligodendrocytes, suggesting that haloperidol affected glucose uptake in these cells (Steiner et al., 2014). In the present study, all the proteins related to glycolysis and gluconeogenesis were increased by haloperidol and decreased by chlorpromazine, whereas none of the atypical antipsychotics used in this study altered the levels of proteins belonging this pathway.

#### 14-3-3 Family

Another set of proteins found at altered levels following chlorpromazine and haloperidol treatments, was proteins from the 14-3-3 family. There are seven known mammalian 14-3-3 isoforms, and 14-3-3 proteins are abundant in the brain, accounting for approximately 1% of the total soluble proteins (Fu et al., 2000). 14-3-3 proteins play fundamental roles in many processes, including the cell cycle, apoptosis, synaptic plasticity, and neuronal differentiation and migration (Fu et al., 2000). In this study, the haloperidol treatment led to decreased levels of 14-3-3 epsilon (YWHAE), and the chlorpromazine treatments induced increased levels of 14-3-3 beta/alpha (YWHAB), 14-3-3 zeta/delta (YWHAZ) and 14-3-3 gamma (YWHAG). Several studies found changes in proteins of the 14-3-3 family in brain tissues from schizophrenia patients (Middleton et al., 2005; Schubert et al., 2015; Qing et al., 2016). Our group found disruption of 14-3-3 signaling in the corpus callosum of patients with schizophrenia (Saia et al., 2015). We also observed that clozapine treatment resulted in increased levels of 14-3-3 protein eta (YWHAH) (Cassoli et al., 2016). However, we did not observe these effects of the atypical antipsychotics used in this study.

### Drug-Specific Differences Triggered by Antipsychotics in the Oligodendrocytes Proteome

#### Chlorpromazine

Neuregulins are member proteins of the epidermal growth factor (EGF) family and are ligands for tyrosine kinases receptors (ErbB family), which play a key role in the development, maintenance, and repair of the nervous system. Recent genetic studies have demonstrated a possible role of neuregulin 1 and its receptor erbB in the pathophysiology of schizophrenia (Hashimoto et al., 2004; Hahn et al., 2006; Avramopoulos, 2017). Moreover, neuregulins play roles in some processes implicated in schizophrenia, such as neuronal migration, neurotransmitter function such as NMDA, GABA, α-7, as well as dopamine and oligodendrocyte biology (Law et al., 2006). Although one study using mRNA expression profiling found no changes in neuregulins (type I, type II, and type III) in the dorsolateral prefrontal cortex of patients with schizophrenia (Hashimoto et al., 2004), another study analyzed mRNA abundance of Neuregulin I (types I-IV) in the hippocampus of patients with schizophrenia and found a variation in the expression of these isoforms (Law et al., 2006). Although neuregulin expression was not affected by chlorpromazine, according to the IPA analysis, some neuregulin-associated proteins, such as HSP90AA1, HSP90B1, MAP2K1, and RPS6 were altered.

#### Haloperidol

A regular side effect of haloperidol treatment and its limitation in the clinic is the extrapyramidal symptoms and tardive dyskinesia. However, the exact pathophysiology of how this drug induces these side effects is not totally clear (Perera et al., 2011). Oxidative stress constitutes a potential pathogenic mechanism that may contribute to movement disturbances, especially to tardive dyskinesia (Perera et al., 2011; Wu et al., 2014; Samad and Haleem, 2017). Recent studies have shown that the repeated administration of haloperidol is able to induce tardive dyskinesia in rats (Samad and Haleem, 2017). Another study using plasma and the enzyme manganese superoxide dismutase (MnSOD) as a biomarker of patients with schizophrenia analyzed the relationship of oxidative stress and tardive dyskinesia. This study suggested that tardive dyskinesia is more severe in patients suffering from oxidative stress (Wu et al., 2014). Furthermore, haloperidol was able to induce oxidative stress, potentially by decreasing the levels of mnSOD and glutathione peroxidase (GPx) in rats (Perera et al., 2011). Here, we do not observed alterations in levels of mnSOD or GPx, but the levels of other glutathione proteins were altered in oligodendrocytes treated with haloperidol, indicating a possible increase of oxidative stress in these cells (Figure 3). However, further investigations are needed to understand possible outcomes for white matter in the schizophrenia.

#### Quetiapine

Quetiapine has higher affinity for serotonin 5HT2 receptors than D2 dopamine receptors (Leucht et al., 1999). Here, compared to the other drugs, quetiapine altered fewer proteins (19). Interestingly, we also observed this profile in the proteome and lipidome from plasma samples of schizophrenia patients (unpublished data). Leucht et al. (2015) recently presented a study using an approach for dose equivalence of second-generation antipsychotic drugs for olanzapine 1 mg/day. The dose equivalent to 1 mg/d olanzapine were quetiapine 32.3 mg/day. In our study we used the same dose for all drugs. In this regard, we hypothesized that a higher concentration of quetiapine would be needed to have a significant response. Although IPA analysis showed associations with 17 canonical pathways, we did not have a ratio higher than 0.06. In this way, there is still little information in our data about these pathways for supporting any relation of these to schizophrenia. However, these results may be relevant to a more specific mechanism of action of quetiapine, although is not clear whether these are side effect or efficacy-related.

#### Risperidone

Purine and pyrimidine nucleotides are essential for nucleic acid synthesis, providing energy and contributing for many other fundamental processes in the cells (Handford et al., 2006). The purinergic system is not only present in neurons in CNS, but studies have been shown that some purines have specialized roles in glial cells (Handford et al., 2006). Purinergic signaling in glial cells regulates proliferation, motility, survival, differentiation, myelination and participate in the communication between neurons and glial cells (Chadwick and Goode, 2008). The purinergic hypothesis of schizophrenia is based on that a dysfunction of purinergic signaling can be associated with many aspects of the pathology (Inoue et al., 1996; Lara and Souza, 2000). A meta-analysis showed improvement in the positive symptoms and slightly important progressing in the negative symptoms of patients with purinergic modulators (Hirota and Kishi, 2013). Here, the treatment with risperidone altered some proteins belonging to the pathway purine nucleotides *de novo* biosynthesis II. Thus, these data indicate that one of the mechanisms of action of risperidone may be to modulate the purinergic system, which would explain its effectiveness in the treatment of schizophrenia.

## Conclusion

Considering the results discussed above, this study has led to identification of some pathways and proteins that can be modulated by antipsychotics in oligodendrocytes. It was possible to observe that risperidone had more pathways in common with chlorpromazine and haloperidol than with quetiapine. It is known that different doses of this drug can lead to different responses in patients, with the capability of behaving as a typical antipsychotic rather than an atypical one. Thus, we suggest that the dose used in the present study resulted in a closer response to typical antipsychotics. In addition, chlorpromazine appears to act by increasing protein levels, whereas haloperidol has the opposite effect. Although both are first-generation antipsychotics, they appear to have different mechanisms of action in oligodendrocytes. Finally, quetiapine altered fewer proteins compared to the other three drugs. Although *in vitro* studies have numerous limitations and may be distant from the *in vivo* scenario, our results provide detailed information on proteins and pathways modulated by the antipsychotics studied here and show the importance of studying cell types other than neurons to fully comprehend the molecular changes involved in both the disease and treatment response. Furthermore, antipsychotics can modulate important functions of oligodendrocytes and this deserves further investigation, since in schizophrenia was found dysregulations in the white matter and alterations in the functions of these cells.

## Data Availability

The datasets generated for this study can be found in the ProteomeXchange http://www.proteomexchange.org/Project accession: PXD008892.

## Author Contributions

CB-T conceived and designed the study and wrote the first draft and final version of the manuscript. JC helped in experimental design and performed the mass spectrometry experiments. VdA helped in data interpretation and manuscript revision. DM-d-S conceived, supervised, and finalized the manuscript.

## Conflict of Interest Statement

The authors declare that the research was conducted in the absence of any commercial or financial relationships that could be construed as a potential conflict of interest.

## Footnotes

1 www.ingenuity.com

## Supplementary Material

Figure S1 Representative images of MO3.13 cells treated for 8 h with vehicle solution (DMSO; **A**) and quetiapine 50 μM **(B)** stained for cytochrome C (green)/Dapi (blue). **(C)** Statistic chart showing normalized intensity fluorescence for cytochrome C for each treatment (^∗^*P* < 0.05). Scale bars = 200 μm.

Table S1 Proteins affected by chlorpromazine treatment.

Table S2 Proteins affected by haloperidol treatment.

Table S3 Ingenuity canonical pathway analysis for oligodendrocyte treated with first generation antipsychotics.

Table S4 Proteins affected by quetiapine treatment.

Table S5 Proteins affected by risperidone treatment.

Table S6 Ingenuity canonical pathway analysis for oligodendrocyte treated with second generation antipsychotics.
