# Corrigendum: Biochemical Pathways Triggered by Antipsychotics in Human Oligodendrocytes: Potential of Discovering New Treatment Targets

**DOI:** 10.3389/fphar.2019.00344

**Published:** 2019-04-09

**Authors:** Caroline Brandão-Teles, Valéria de Almeida, Juliana S. Cassoli, Daniel Martins-de-Souza

**Affiliations:** ^1^Laboratory of Neuroproteomics, Department of Biochemistry and Tissue Biology, Institute of Biology, University of Campinas, Campinas, Brazil; ^2^Faculdade de Palmas, Palmas, Brazil; ^3^UNICAMP's Neurobiology Center, Campinas, Brazil; ^4^Instituto Nacional de Biomarcadores em Neuropsiquiatria, Conselho Nacional de Desenvolvimento Científico e Tecnológico, São Paulo, Brazil

**Keywords:** schizophrenia, mechanism of action, proteomics, biomarkers, chlorpromazine, haloperidol, quetiapine, risperidone

The title of the article has been changed from “Oligodendrocytes: Potential of Discovering New Treatment Targets” to “Biochemical Pathways Triggered by Antipsychotics in Human Oligodendrocytes: Potential of Discovering New Treatment Targets.”

The authors apologize for this error and state that this does not change the scientific conclusions of the article in any way. The original article has been updated.

