# Supplementary figures and images for: Biochemical Pathways Triggered by Antipsychotics in Human Oligodendrocytes: Potential of Discovering New Treatment Targets
Source: Front Pharmacol. 2019 Mar 5;10:186. doi: 10.3389/fphar.2019.00186 (PMC6411851; doi:10.3389/fphar.2019.00186)

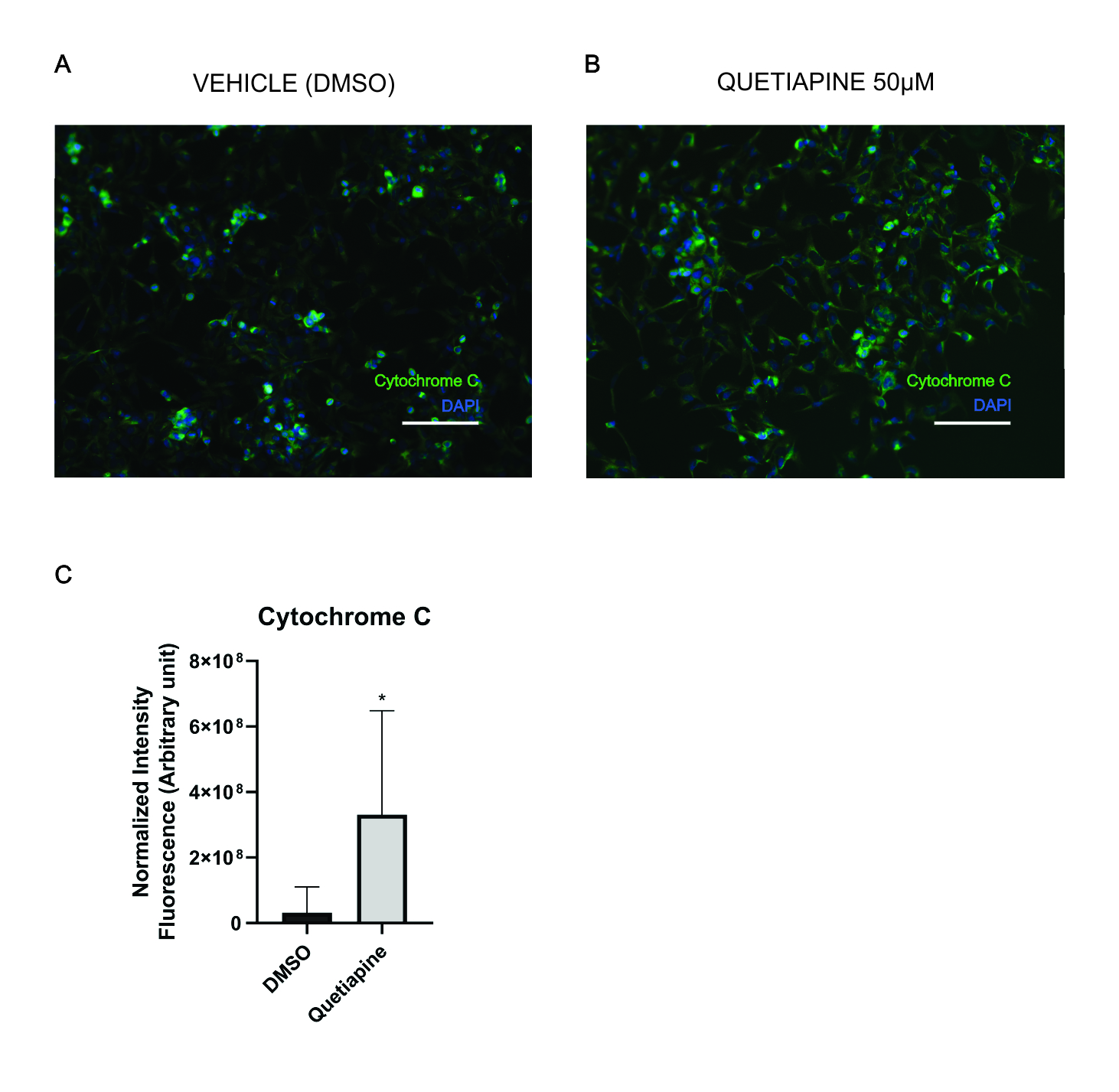

Supplement: Figure S1 — Representative images of MO3.13 cells treated for 8 h with vehicle solution (DMSO; A) and quetiapine 50 μM (B) stained for cytochrome C (green)/Dapi (blue). (C) Statistic chart showing normalized intensity fluorescence for cytochrome C for each treatment (∗P < 0.05). Scale bars = 200 μm. [file Image_1.JPEG]
