# Supplementary material for: Biochemical Pathways Triggered by Antipsychotics in Human Oligodendrocytes: Potential of Discovering New Treatment Targets
Source: Front Pharmacol. 2019 Mar 5;10:186. doi: 10.3389/fphar.2019.00186 (PMC6411851; doi:10.3389/fphar.2019.00186)
Supplement: Table S1 — Proteins affected by chlorpromazine treatment. [file Table_1.DOCX]

| Table 1: Proteins affected by chlorpromazine treatment | | | | |
| --- | --- | --- | --- | --- |
| *Accession* | *Gene* | *Anova (p)* | *Log2 FC* | *Protein* |
| O00469 | PLOD2 | 6,27251E-05 | -2,668795015 | Procollagen-lysine_2-oxoglutarate 5-dioxygenase 2 |
| Q96GS4 | BORCS6 | 0,043699905 | -2,082038911 | BLOC-1-related complex subunit 6 |
| Q5BJD5 | TMEM41B | 0,017428292 | -1,900290573 | Transmembrane protein 41B |
| P01023 | A2M | 0,012003471 | -1,618107167 | Alpha-2-macroglobulin |
| P02771 | AFP | 0,024465155 | -1,459135501 | Alpha-fetoprotein |
| Q9Y6M1 | IGF2BP2 | 0,044416582 | -1,313192827 | Insulin-like growth factor 2 mRNA-binding protein 2 |
| P09104 | ENO2 | 0,027071829 | -1,17192323 | Gamma-enolase |
| P13995 | MTHFD2 | 0,012413945 | -1,152560325 | Bifunctional methylenetetrahydrofolate dehydrogenase/cyclohydrolase_ mitochondrial |
| P36871 | PGM1 | 0,028314369 | -1,115732471 | Phosphoglucomutase-1 |
| O60568 | PLOD3 | 0,000138087 | -1,091092391 | Procollagen-lysine_2-oxoglutarate 5-dioxygenase 3 |
| Q5VTE0 | EEF1A1P5 | 0,01650851 | -1,016183384 | Putative elongation factor 1-alpha-like 3 |
| P51991 | HNRNPA3 | 0,030022286 | -0,978906432 | Heterogeneous nuclear ribonucleoprotein A3 |
| Q9NRY5 | FAM114A2 | 0,003106202 | -0,960258032 | Protein FAM114A2 |
| Q04637 | EIF4G1 | 0,02549273 | -0,943738771 | Eukaryotic translation initiation factor 4 gamma 1 |
| Q9NVP1 | DDX18 | 0,012906035 | -0,940584442 | ATP-dependent RNA helicase DDX18 |
| O00425 | IGF2BP3 | 0,028068435 | -0,939812975 | Insulin-like growth factor 2 mRNA-binding protein 3 |
| P11021 | HSPA5 | 0,027574404 | -0,896792219 | Endoplasmic reticulum chaperone BiP |
| Q02543 | RPL18A | 0,000334024 | -0,856228919 | 60S ribosomal protein L18a |
| P62834 | RAP1A | 0,01916093 | -0,855752453 | Ras-related protein Rap-1A |
| P30044 | PRDX5 | 0,005589866 | -0,8465209 | Peroxiredoxin-5_ mitochondrial |
| Q32P28 | P3H1 | 0,032925425 | -0,846078472 | Prolyl 3-hydroxylase 1 |
| Q92499 | DDX1 | 0,001653289 | -0,843955244 | ATP-dependent RNA helicase DDX1 |
| Q96PU8 | QKI | 0,004787067 | -0,809506185 | Protein quaking |
| Q9BPX7 | C7orf25 | 0,002795237 | -0,785299459 | UPF0415 protein C7orf25 |
| Q96BM9 | ARL8A | 0,011670921 | -0,780658932 | ADP-ribosylation factor-like protein 8A |
| P08195 | SLC3A2 | 0,03487127 | -0,773955598 | 4F2 cell-surface antigen heavy chain |
| P26373 | RPL13 | 0,013629111 | -0,768646553 | 60S ribosomal protein L13 |
| P0CG38 | POTEI | 0,007329629 | -0,728861213 | POTE ankyrin domain family member I |
| Q02750 | MAP2K1 | 0,018208531 | -0,715773581 | Dual specificity mitogen-activated protein kinase kinase 1 |
| P55087 | AQP4 | 0,025755744 | -0,668786801 | Aquaporin-4 |
| Q9Y678 | COPG1 | 0,00578016 | -0,668234283 | Coatomer subunit gamma-1 |
| P31150 | GDI1 | 0,01715596 | -0,653774264 | Rab GDP dissociation inhibitor alpha |
| P50991 | CCT4 | 0,005577181 | -0,638058288 | T-complex protein 1 subunit delta |
| Q9UHD8 | SEPT9 | 0,018173191 | -0,608975093 | Septin-9 |
| P48047 | ATP5O | 1,92417E-06 | -0,572438091 | ATP synthase subunit O_ mitochondrial |
| Q9H773 | DCTPP1 | 0,006579955 | -0,563745472 | dCTP pyrophosphatase 1 |
| Q9NR30 | DDX21 | 0,000670986 | -0,558750927 | Nucleolar RNA helicase 2 |
| P15927 | RPA2 | 0,024351328 | -0,555162149 | Replication protein A 32 kDa subunit |
| P33176 | KIF5B | 0,003110982 | -0,537790222 | Kinesin-1 heavy chain |
| Q14240 | EIF4A2 | 0,001604233 | -0,532657137 | Eukaryotic initiation factor 4A-II |
| P00387 | CYB5R3 | 0,013748803 | -0,518216026 | NADH-cytochrome b5 reductase 3 |
| O43175 | PHGDH | 0,004512993 | -0,51463721 | D-3-phosphoglycerate dehydrogenase |
| P61289 | PSME3 | 0,016038533 | -0,511386119 | Proteasome activator complex subunit 3 |
| Q99733 | NAP1L4 | 0,002866929 | -0,500615319 | Nucleosome assembly protein 1-like 4 |
| P54652 | HSPA2 | 0,007766833 | -0,49889119 | Heat shock-related 70 kDa protein 2 |
| P35659 | DEK | 0,044320192 | -0,490394913 | Protein DEK |
| P0CG39 | POTEJ | 0,030038941 | -0,462429115 | POTE ankyrin domain family member J |
| P30613 | PKLR | 0,026437302 | -0,438935679 | Pyruvate kinase PKLR |
| Q15287 | RNPS1 | 0,027453359 | -0,428631928 | RNA-binding protein with serine-rich domain 1 |
| O00487 | PSMD14 | 0,027049322 | -0,423212423 | 26S proteasome non-ATPase regulatory subunit 14 |
| Q96Q15 | SMG1 | 0,035384772 | -0,384557458 | Serine/threonine-protein kinase SMG1 |
| P15311 | EZR | 0,003024012 | -0,377157784 | Ezrin |
| P07237 | P4HB | 0,010169951 | -0,365026437 | Protein disulfide-isomerase |
| E9PAV3 | NACA | 0,01263982 | -0,363420996 | Nascent polypeptide-associated complex subunit alpha_ muscle-specific form |
| Q13435 | SF3B2 | 0,015502287 | -0,36009282 | Splicing factor 3B subunit 2 |
| P35249 | RFC4 | 0,003639834 | -0,340714706 | Replication factor C subunit 4 |
| P04792 | HSPB1 | 0,013751098 | -0,322126032 | Heat shock protein beta-1 |
| Q9NZI8 | IGF2BP1 | 0,049658396 | -0,30931418 | Insulin-like growth factor 2 mRNA-binding protein 1 |
| Q5BKZ1 | ZNF326 | 0,011335521 | -0,304449855 | DBIRD complex subunit ZNF326 |
| Q9H299 | SH3BGRL3 | 0,028267435 | -0,302171499 | SH3 domain-binding glutamic acid-rich-like protein 3 |
| P12277 | CKB | 0,022874261 | -0,29424173 | Creatine kinase B-type |
| Q9Y230 | RUVBL2 | 0,009582003 | -0,292895014 | RuvB-like 2 |
| P67809 | YBX1 | 0,033112934 | -0,281964717 | Nuclease-sensitive element-binding protein 1 |
| Q14847 | LASP1 | 0,010348544 | -0,260428953 | LIM and SH3 domain protein 1 |
| O15067 | PFAS | 0,021670953 | -0,219533707 | Phosphoribosylformylglycinamidine synthase |
| P07205 | PGK2 | 0,014965203 | -0,206924614 | Phosphoglycerate kinase 2 |
| P49321 | NASP | 0,016002652 | -0,179837357 | Nuclear autoantigenic sperm protein |
| P42771 | CDKN2A | 0,03741658 | 0,209768394 | Cyclin-dependent kinase inhibitor 2A |
| P62701 | RPS4X | 0,020674988 | 0,272145851 | 40S ribosomal protein S4_ X isoform |
| Q15003 | NCAPH | 0,019287297 | 0,282836552 | Condensin complex subunit 2 |
| P07910 | HNRNPC | 0,034003417 | 0,284351552 | Heterogeneous nuclear ribonucleoproteins C1/C2 |
| P07900 | HSP90AA1 | 0,015098145 | 0,307142599 | Heat shock protein HSP 90-alpha |
| P14550 | AKR1A1 | 0,007666224 | 0,32949846 | Alcohol dehydrogenase [NADP(+)] |
| P39023 | RPL3 | 0,023463679 | 0,336383284 | 60S ribosomal protein L3 |
| P17066 | HSPA6 | 0,017205515 | 0,36214806 | Heat shock 70 kDa protein 6 |
| P84103 | SRSF3 | 0,014777197 | 0,372461601 | Serine/arginine-rich splicing factor 3 |
| O43809 | NUDT21 | 0,002187968 | 0,38851393 | Cleavage and polyadenylation specificity factor subunit 5 |
| P68400 | CSNK2A1 | 0,048087978 | 0,409792367 | Casein kinase II subunit alpha |
| O14818 | PSMA7 | 0,018569455 | 0,410185692 | Proteasome subunit alpha type-7 |
| P12829 | MYL4 | 0,026179561 | 0,423742482 | Myosin light chain 4 |
| P18621 | RPL17 | 0,039348916 | 0,431657235 | 60S ribosomal protein L17 |
| P38159 | RBMX | 0,03256908 | 0,437651854 | RNA-binding motif protein_ X chromosome |
| P46781 | RPS9 | 0,023119916 | 0,438668961 | 40S ribosomal protein S9 |
| Q9UMR2 | DDX19B | 0,025070759 | 0,447386531 | ATP-dependent RNA helicase DDX19B |
| Q32P51 | HNRNPA1L2 | 0,033855359 | 0,452562622 | Heterogeneous nuclear ribonucleoprotein A1-like 2 |
| Q09028 | RBBP4 | 0,01479412 | 0,454443841 | Histone-binding protein RBBP4 |
| P52209 | PGD | 0,006787636 | 0,459529491 | 6-phosphogluconate dehydrogenase_ decarboxylating |
| Q01082 | SPTBN1 | 0,018304177 | 0,463114581 | Spectrin beta chain_ non-erythrocytic 1 |
| P55209 | NAP1L1 | 0,035136582 | 0,470506744 | Nucleosome assembly protein 1-like 1 |
| P20340 | RAB6A | 0,049022361 | 0,474347609 | Ras-related protein Rab-6A |
| P62318 | SNRPD3 | 0,036360794 | 0,479780263 | Small nuclear ribonucleoprotein Sm D3 |
| P62753 | RPS6 | 0,003002027 | 0,48168443 | 40S ribosomal protein S6 |
| Q9UQ80 | PA2G4 | 0,041276272 | 0,487586702 | Proliferation-associated protein 2G4 |
| P25705 | ATP5A1 | 0,023691001 | 0,488907959 | ATP synthase subunit alpha_ mitochondrial |
| P62424 | RPL7A | 0,040569063 | 0,490277147 | 60S ribosomal protein L7a |
| Q5TZA2 | CROCC | 0,007416116 | 0,503071639 | Rootletin |
| Q15366 | PCBP2 | 0,025242014 | 0,503810431 | Poly(rC)-binding protein 2 |
| P22392 | NME2 | 0,035325585 | 0,508508348 | Nucleoside diphosphate kinase B |
| P14625 | HSP90B1 | 0,034463 | 0,5093106 | Endoplasmin |
| P30405 | PPIF | 0,049096513 | 0,510292469 | Peptidyl-prolyl cis-trans isomerase F_ mitochondrial |
| P61758 | VBP1 | 0,015066227 | 0,512898032 | Prefoldin subunit 3 |
| P43487 | RANBP1 | 0,041064805 | 0,525804046 | Ran-specific GTPase-activating protein |
| Q9UNM6 | PSMD13 | 0,019861342 | 0,536907954 | 26S proteasome non-ATPase regulatory subunit 13 |
| Q9Y266 | NUDC | 0,011909919 | 0,541508097 | Nuclear migration protein nudC |
| P62195 | PSMC5 | 0,01902642 | 0,545419598 | 26S proteasome regulatory subunit 8 |
| P34897 | SHMT2 | 0,003831137 | 0,54899855 | Serine hydroxymethyltransferase_ mitochondrial |
| P07355 | ANXA2 | 0,037300385 | 0,562972296 | Annexin A2 |
| P06493 | CDK1 | 0,034956784 | 0,566796139 | Cyclin-dependent kinase 1 |
| P23921 | RRM1 | 0,04574874 | 0,606908218 | Ribonucleoside-diphosphate reductase large subunit |
| Q6ZMU5 | TRIM72 | 0,024294712 | 0,615650193 | Tripartite motif-containing protein 72 |
| O14737 | PDCD5 | 0,001526171 | 0,63039907 | Programmed cell death protein 5 |
| P26583 | HMGB2 | 0,006124628 | 0,634589893 | High mobility group protein B2 |
| P62899 | RPL31 | 0,014268995 | 0,636698137 | 60S ribosomal protein L31 |
| Q13526 | PIN1 | 0,043810329 | 0,639989104 | Peptidyl-prolyl cis-trans isomerase NIMA-interacting 1 |
| P07737 | PFN1 | 0,044960627 | 0,645300596 | Profilin-1 |
| P53999 | SUB1 | 0,005540474 | 0,646023571 | Activated RNA polymerase II transcriptional coactivator p15 |
| P62277 | RPS13 | 0,016180078 | 0,65673139 | 40S ribosomal protein S13 |
| Q9Y277 | VDAC3 | 0,046601643 | 0,663171555 | Voltage-dependent anion-selective channel protein 3 |
| Q9UKI2 | CDC42EP3 | 0,001440013 | 0,666198222 | Cdc42 effector protein 3 |
| P31946 | YWHAB | 0,021234528 | 0,671185536 | 14-3-3 protein beta/alpha |
| P61604 | HSPE1 | 0,016090944 | 0,671787286 | 10 kDa heat shock protein_ mitochondrial |
| P05388 | RPLP0 | 0,013676757 | 0,673247883 | 60S acidic ribosomal protein P0 |
| Q9Y285 | FARSA | 0,003882837 | 0,674379745 | Phenylalanine--tRNA ligase alpha subunit |
| P61978 | HNRNPK | 0,026709252 | 0,677918586 | Heterogeneous nuclear ribonucleoprotein K |
| Q7L1Q6 | BZW1 | 0,003654733 | 0,681268843 | Basic leucine zipper and W2 domain-containing protein 1 |
| O43143 | DHX15 | 0,036437569 | 0,686997954 | Pre-mRNA-splicing factor ATP-dependent RNA helicase DHX15 |
| P18206 | VCL | 0,048968394 | 0,690242215 | Vinculin |
| Q9BTV4 | TMEM43 | 0,012518785 | 0,692321195 | Transmembrane protein 43 |
| P09874 | PARP1 | 0,020616391 | 0,69251766 | Poly [ADP-ribose] polymerase 1 |
| P62750 | RPL23A | 0,012830044 | 0,695323608 | 60S ribosomal protein L23a |
| P05976 | MYL1 | 0,003009757 | 0,709566408 | Myosin light chain 1/3_ skeletal muscle isoform |
| Q96K17 | BTF3L4 | 0,008845508 | 0,722909718 | Transcription factor BTF3 homolog 4 |
| Q9H0D6 | XRN2 | 0,000192688 | 0,735849744 | 5'-3' exoribonuclease 2 |
| Q03113 | GNA12 | 0,03535324 | 0,746890956 | Guanine nucleotide-binding protein subunit alpha-12 |
| A0A0B4J2A2 | PPIAL4C | 0,019114904 | 0,752318768 | Peptidyl-prolyl cis-trans isomerase A-like 4C |
| P30048 | PRDX3 | 0,041661469 | 0,756776785 | Thioredoxin-dependent peroxide reductase_ mitochondrial |
| O14980 | XPO1 | 0,008517546 | 0,768477725 | Exportin-1 |
| Q9BYZ2 | LDHAL6B | 0,011423792 | 0,769299466 | L-lactate dehydrogenase A-like 6B |
| P13804 | ETFA | 0,030982831 | 0,807106517 | Electron transfer flavoprotein subunit alpha_ mitochondrial |
| Q99497 | PARK7 | 0,013999444 | 0,814444957 | Protein/nucleic acid deglycase DJ-1 |
| Q13242 | SRSF9 | 0,006818283 | 0,819352708 | Serine/arginine-rich splicing factor 9 |
| P06744 | GPI | 0,004489779 | 0,827491606 | Glucose-6-phosphate isomerase |
| O00303 | EIF3F | 0,048523596 | 0,829694938 | Eukaryotic translation initiation factor 3 subunit F |
| O75821 | EIF3G | 0,020356249 | 0,835022986 | Eukaryotic translation initiation factor 3 subunit G |
| P15531 | NME1 | 0,00342768 | 0,835474365 | Nucleoside diphosphate kinase A |
| P61619 | SEC61A1 | 0,045903341 | 0,836243578 | Protein transport protein Sec61 subunit alpha isoform 1 |
| P09382 | LGALS1 | 0,001046272 | 0,845683985 | Galectin-1 |
| Q92928 | RAB1C | 0,004188545 | 0,85836332 | Putative Ras-related protein Rab-1C |
| P63104 | YWHAZ | 0,043216774 | 0,867581241 | 14-3-3 protein zeta/delta |
| Q08257 | CRYZ | 0,041671035 | 0,877380096 | Quinone oxidoreductase |
| O75880 | SCO1 | 0,045246129 | 0,893453761 | Protein SCO1 homolog_ mitochondrial |
| Q969Z4 | RELT | 0,019695018 | 0,897993293 | Tumor necrosis factor receptor superfamily member 19L |
| P32969 | RPL9 | 0,001967062 | 0,901223181 | 60S ribosomal protein L9 |
| P46782 | RPS5 | 0,041296065 | 0,90263504 | 40S ribosomal protein S5 |
| Q8NFC6 | BOD1L1 | 0,001710758 | 0,91231937 | Biorientation of chromosomes in cell division protein 1-like 1 |
| O43242 | PSMD3 | 0,04858537 | 0,932526907 | 26S proteasome non-ATPase regulatory subunit 3 |
| Q9Y281 | CFL2 | 0,009425625 | 0,96424428 | Cofilin-2 |
| P00568 | AK1 | 0,002298673 | 0,96690393 | Adenylate kinase isoenzyme 1 |
| Q15050 | RRS1 | 0,028134207 | 0,977688158 | Ribosome biogenesis regulatory protein homolog |
| P68431 | HIST1H3A | 0,041334428 | 0,997736332 | Histone H3.1 |
| P32119 | PRDX2 | 0,006596926 | 1,002729042 | Peroxiredoxin-2 |
| O43684 | BUB3 | 0,00128696 | 1,047822442 | Mitotic checkpoint protein BUB3 |
| P61026 | RAB10 | 0,012794043 | 1,068152138 | Ras-related protein Rab-10 |
| Q8IUE6 | HIST2H2AB | 0,017608115 | 1,069888148 | Histone H2A type 2-B |
| Q9Y6V0 | PCLO | 0,005230772 | 1,076807844 | Protein piccolo |
| P05387 | RPLP2 | 0,010389586 | 1,078579687 | 60S acidic ribosomal protein P2 |
| P46087 | NOP2 | 0,009943347 | 1,095768706 | Probable 28S rRNA (cytosine(4447)-C(5))-methyltransferase |
| P49588 | AARS | 0,000414221 | 1,125719114 | Alanine--tRNA ligase_ cytoplasmic |
| Q9H4M9 | EHD1 | 0,04053322 | 1,127389654 | EH domain-containing protein 1 |
| P31949 | S100A11 | 0,035581225 | 1,131964493 | Protein S100-A11 |
| Q96B54 | ZNF428 | 0,012839904 | 1,137462722 | Zinc finger protein 428 |
| Q16774 | GUK1 | 0,012562858 | 1,141781432 | Guanylate kinase |
| P27797 | CALR | 0,003132452 | 1,144360885 | Calreticulin |
| P08708 | RPS17 | 0,020557442 | 1,17780381 | 40S ribosomal protein S17 |
| P61981 | YWHAG | 0,00033359 | 1,190657988 | 14-3-3 protein gamma |
| Q9NQA5 | TRPV5 | 0,020670883 | 1,197839467 | Transient receptor potential cation channel subfamily V member 5 |
| Q9H488 | POFUT1 | 0,032379852 | 1,20157681 | GDP-fucose protein O-fucosyltransferase 1 |
| O95373 | IPO7 | 0,002038105 | 1,202121972 | Importin-7 |
| P49419 | ALDH7A1 | 0,020139192 | 1,2113822 | Alpha-aminoadipic semialdehyde dehydrogenase |
| Q9BRA2 | TXNDC17 | 0,001549405 | 1,215923238 | Thioredoxin domain-containing protein 17 |
| Q13867 | BLMH | 0,011165135 | 1,261739619 | Bleomycin hydrolase |
| Q9BWM7 | SFXN3 | 0,001754509 | 1,269952262 | Sideroflexin-3 |
| Q8N485 | LIX1 | 0,033479225 | 1,27403635 | Protein limb expression 1 homolog |
| Q9Y2B0 | CNPY2 | 0,026546479 | 1,287306929 | Protein canopy homolog 2 |
| O00560 | SDCBP | 0,001278723 | 1,513818501 | Syntenin-1 |
| Q9NY65 | TUBA8 | 0,008676838 | 1,583209618 | Tubulin alpha-8 chain |
| P52597 | HNRNPF | 0,01038384 | 1,603266301 | Heterogeneous nuclear ribonucleoprotein F |
| Q9BRK5 | SDF4 | 0,012422615 | 1,650927651 | 45 kDa calcium-binding protein |
| P28838 | LAP3 | 0,000564942 | 1,935740919 | Cytosol aminopeptidase |
| Q9UKM9 | RALY | 0,04324471 | 2,061836122 | RNA-binding protein Raly |
| Q96QV6 | HIST1H2AA | 0,000611197 | 2,199893859 | Histone H2A type 1-A |
| Q7Z7K6 | CENPV | 0,015108653 | 2,314661434 | Centromere protein V |
| P30049 | ATP5D | 0,003067757 | 2,418822597 | ATP synthase subunit delta_ mitochondrial |
| Q14444 | CAPRIN1 | 0,004712272 | 2,588915352 | Caprin-1 |
| Q8NFI3 | ENGASE | 0,000376883 | 2,658398622 | Cytosolic endo-beta-N-acetylglucosaminidase |
