# Supplementary material for: Biochemical Pathways Triggered by Antipsychotics in Human Oligodendrocytes: Potential of Discovering New Treatment Targets
Source: Front Pharmacol. 2019 Mar 5;10:186. doi: 10.3389/fphar.2019.00186 (PMC6411851; doi:10.3389/fphar.2019.00186)
Supplement: Table S2 — Proteins affected by haloperidol treatment. [file Table_2.DOCX]

| Table 2 - Proteins affected by haloperidol treatment | | | | |
| --- | --- | --- | --- | --- |
| *Accession* | *Gene* | *Anova (p)* | *Log 2 FC* | *Protein* |
| P04183 | TK1 | 6,53318E-05 | -8,16133 | Thymidine kinase_ cytosolic |
| Q99661 | KIF2C | 2,27927E-05 | -4,68488 | Kinesin-like protein KIF2C |
| Q8TAT5 | NEIL3 | 0,002800123 | -4,53237 | Endonuclease 8-like 3 |
| Q15286 | RAB35 | 0,000993361 | -3,5416 | Ras-related protein Rab-35 |
| Q86SE5 | RALYL | 0,000390954 | -3,41273 | RNA-binding Raly-like protein |
| Q15389 | ANGPT1 | 0,00926775 | -3,38971 | Angiopoietin-1 |
| Q562R1 | ACTBL2 | 0,002000184 | -2,90083 | Beta-actin-like protein 2 |
| P24666 | ACP1 | 0,001264289 | -2,89522 | Low molecular weight phosphotyrosine protein phosphatase |
| Q96FW1 | OTUB1 | 0,010810601 | -2,80219 | Ubiquitin thioesterase OTUB1 |
| Q8NAV2 | C8orf58 | 0,007469125 | -2,76899 | Uncharacterized protein C8orf58 |
| P05204 | HMGN2 | 0,00173888 | -2,52179 | Non-histone chromosomal protein HMG-17 |
| Q96L33 | RHOV | 0,00012961 | -2,49372 | Rho-related GTP-binding protein RhoV |
| P55795 | HNRNPH2 | 0,003909737 | -2,47724 | Heterogeneous nuclear ribonucleoprotein H2 |
| Q13347 | EIF3I | 0,00141843 | -2,439 | Eukaryotic translation initiation factor 3 subunit I |
| Q01082 | SPTBN1 | 0,018957844 | -2,35302 | Spectrin beta chain_ non-erythrocytic 1 |
| P63220 | RPS21 | 0,002938182 | -2,30019 | 40S ribosomal protein S21 |
| Q9Y383 | LUC7L2 | 0,028332247 | -2,2198 | Putative RNA-binding protein Luc7-like 2 |
| P25398 | RPS12 | 9,2337E-05 | -2,20436 | 40S ribosomal protein S12 |
| O75526 | RBMXL2 | 0,001683335 | -2,07337 | RNA-binding motif protein_ X-linked-like-2 |
| O95273 | CCNDBP1 | 2,4636E-05 | -2,05909 | Cyclin-D1-binding protein 1 |
| Q4VX62 | C6orf99 | 0,001168359 | -1,93505 | Putative uncharacterized protein C6orf99 |
| Q03252 | LMNB2 | 0,013738738 | -1,90825 | Lamin-B2 |
| P11021 | HSPA5 | 0,02237828 | -1,8544 | Endoplasmic reticulum chaperone BiP |
| P50990 | CCT8 | 0,001655371 | -1,80349 | T-complex protein 1 subunit theta |
| P17980 | PSMC3 | 0,003267125 | -1,77414 | 26S proteasome regulatory subunit 6A |
| Q9BRP8 | PYM1 | 0,02202288 | -1,74786 | Partner of Y14 and mago |
| Q8WWM7 | ATXN2L | 0,012901482 | -1,69178 | Ataxin-2-like protein |
| Q01105 | SET | 2,262E-05 | -1,64751 | Protein SET |
| P0DME0 | SETSIP | 2,26201E-05 | -1,64751 | Protein SETSIP |
| P08758 | ANXA5 | 0,045402601 | -1,60226 | Annexin A5 |
| A6NH52 | TVP23A | 0,021659615 | -1,5548 | Golgi apparatus membrane protein TVP23 homolog A |
| A6NGU5 | GGT3P | 0,015036467 | -1,54208 | Putative glutathione hydrolase 3 proenzyme |
| P57721 | PCBP3 | 0,018089009 | -1,53833 | Poly(rC)-binding protein 3 |
| Q9NSB2 | KRT84 | 0,000494936 | -1,49219 | Keratin_ type II cuticular Hb4 |
| P21333 | FLNA | 0,015694854 | -1,45341 | Filamin-A |
| P02771 | AFP | 0,000134746 | -1,43067 | Alpha-fetoprotein |
| P05452 | CLEC3B | 0,00055929 | -1,41248 | Tetranectin |
| Q15365 | PCBP1 | 0,018992128 | -1,34494 | Poly(rC)-binding protein 1 |
| P48741 | HSPA7 | 3,56165E-05 | -1,31769 | Putative heat shock 70 kDa protein 7 |
| P09382 | LGALS1 | 0,048244098 | -1,29541 | Galectin-1 |
| Q15819 | UBE2V2 | 0,028055439 | -1,28047 | Ubiquitin-conjugating enzyme E2 variant 2 |
| Q9Y3I0 | RTCB | 0,009717016 | -1,22408 | tRNA-splicing ligase RtcB homolog |
| P35579 | MYH9 | 0,013526604 | -1,22275 | Myosin-9 |
| P14314 | PRKCSH | 0,012661987 | -1,1138 | Glucosidase 2 subunit beta |
| P49327 | FASN | 0,014637574 | -1,08608 | Fatty acid synthase |
| Q9BUF5 | TUBB6 | 3,11789E-08 | -1,08026 | Tubulin beta-6 chain |
| O75531 | BANF1 | 0,01557908 | -1,051 | Barrier-to-autointegration factor |
| Q86V81 | ALYREF | 0,042444786 | -1,04188 | THO complex subunit 4 |
| Q99961 | SH3GL1 | 0,01127423 | -1,02979 | Endophilin-A2 |
| P04350 | TUBB4A | 0,001845832 | -0,98283 | Tubulin beta-4A chain |
| O95372 | LYPLA2 | 0,012817446 | -0,94774 | Acyl-protein thioesterase 2 |
| P0CG38 | POTEI | 0,003471175 | -0,937 | POTE ankyrin domain family member I |
| P68371 | TUBB4B | 0,030373917 | -0,92652 | Tubulin beta-4B chain |
| P62258 | YWHAE | 0,022798387 | -0,90642 | 14-3-3 protein epsilon |
| O75914 | PAK3 | 0,006280701 | -0,90026 | Serine/threonine-protein kinase PAK 3 |
| Q92599 | SEPT8 | 4,9402E-05 | -0,8707 | Septin-8 |
| O94905 | ERLIN2 | 0,017444628 | -0,86877 | Erlin-2 |
| P54652 | HSPA2 | 0,003946017 | -0,86859 | Heat shock-related 70 kDa protein 2 |
| P47756 | CAPZB | 0,003675053 | -0,82611 | F-actin-capping protein subunit beta |
| Q5VZM2 | RRAGB | 0,045662218 | -0,79125 | Ras-related GTP-binding protein B |
| P06493 | CDK1 | 0,000202652 | -0,76158 | Cyclin-dependent kinase 1 |
| Q96C90 | PPP1R14B | 0,019503261 | -0,75672 | Protein phosphatase 1 regulatory subunit 14B |
| P35659 | DEK | 0,000349118 | -0,74973 | Protein DEK |
| P41091 | EIF2S3 | 0,00489425 | -0,74726 | Eukaryotic translation initiation factor 2 subunit 3 |
| P0CW21 | SPART-AS1 | 0,02374797 | -0,74341 | Putative uncharacterized protein SPART-AS1 |
| P61221 | ABCE1 | 2,30809E-05 | -0,74062 | ATP-binding cassette sub-family E member 1 |
| Q9Y678 | COPG1 | 0,008749533 | -0,73963 | Coatomer subunit gamma-1 |
| P49458 | SRP9 | 0,000851938 | -0,73876 | Signal recognition particle 9 kDa protein |
| P20338 | RAB4A | 0,030661496 | -0,72075 | Ras-related protein Rab-4A |
| Q4VXU2 | PABPC1L | 0,004536605 | -0,70814 | Polyadenylate-binding protein 1-like |
| P07237 | P4HB | 0,000696526 | -0,68239 | Protein disulfide-isomerase |
| P54136 | RARS | 0,019579184 | -0,6564 | Arginine--tRNA ligase_ cytoplasmic |
| P26368 | U2AF2 | 0,022798795 | -0,6522 | Splicing factor U2AF 65 kDa subunit |
| O60506 | SYNCRIP | 0,046394184 | -0,64044 | Heterogeneous nuclear ribonucleoprotein Q |
| Q9NVI7 | ATAD3A | 0,003379532 | -0,63586 | ATPase family AAA domain-containing protein 3A |
| P15153 | RAC2 | 0,006650255 | -0,62739 | Ras-related C3 botulinum toxin substrate 2 |
| Q9BQ52 | ELAC2 | 0,046030915 | -0,56511 | Zinc phosphodiesterase ELAC protein 2 |
| Q9NPQ8 | RIC8A | 0,032301067 | -0,56458 | Synembryn-A |
| P02545 | LMNA | 0,014469116 | -0,54815 | Prelamin-A/C |
| O75880 | SCO1 | 0,020764974 | -0,5088 | Protein SCO1 homolog_ mitochondrial |
| P63165 | SUMO1 | 0,006621488 | -0,48727 | Small ubiquitin-related modifier 1 |
| P43243 | MATR3 | 0,014975777 | -0,44833 | Matrin-3 |
| P62191 | PSMC1 | 0,004245959 | -0,44788 | 26S proteasome regulatory subunit 4 |
| P51149 | RAB7A | 0,011928572 | -0,44424 | Ras-related protein Rab-7a |
| O43399 | TPD52L2 | 0,039413967 | -0,4321 | Tumor protein D54 |
| Q6ZU15 | SEPT14 | 0,003376175 | -0,4259 | Septin-14 |
| Q9BS26 | ERP44 | 0,009590424 | -0,41719 | Endoplasmic reticulum resident protein 44 |
| P31930 | UQCRC1 | 0,00019522 | -0,40471 | Cytochrome b-c1 complex subunit 1_ mitochondrial |
| Q9NVP1 | DDX18 | 0,009361504 | -0,40454 | ATP-dependent RNA helicase DDX18 |
| P07910 | HNRNPC | 0,006934679 | -0,39652 | Heterogeneous nuclear ribonucleoproteins C1/C2 |
| Q6ZMR3 | LDHAL6A | 0,001051038 | -0,37356 | L-lactate dehydrogenase A-like 6A |
| P62917 | RPL8 | 0,025840723 | -0,36919 | 60S ribosomal protein L8 |
| P37837 | TALDO1 | 0,000252798 | -0,35287 | Transaldolase |
| P29692 | EEF1D | 0,002058849 | -0,32476 | Elongation factor 1-delta |
| Q92901 | RPL3L | 0,014777462 | -0,31735 | 60S ribosomal protein L3-like |
| P61586 | RHOA | 0,004833691 | -0,28683 | Transforming protein RhoA |
| Q9UNF1 | MAGED2 | 0,00079222 | -0,25498 | Melanoma-associated antigen D2 |
| P14550 | AKR1A1 | 0,02751437 | -0,23188 | Alcohol dehydrogenase [NADP(+)] |
| P22626 | HNRNPA2B1 | 0,043062831 | -0,22586 | Heterogeneous nuclear ribonucleoproteins A2/B1 |
| Q9P2R7 | SUCLA2 | 0,025373039 | -0,17523 | Succinate--CoA ligase [ADP-forming] subunit beta_ mitochondrial |
| Q5JTV8 | TOR1AIP1 | 0,022773047 | 0,146104 | Torsin-1A-interacting protein 1 |
| P09429 | HMGB1 | 0,018896255 | 0,212061 | High mobility group protein B1 |
| P51665 | PSMD7 | 0,009671508 | 0,219646 | 26S proteasome non-ATPase regulatory subunit 7 |
| P05455 | SSB | 0,03898406 | 0,260492 | Lupus La protein |
| P60900 | PSMA6 | 0,007829226 | 0,272068 | Proteasome subunit alpha type-6 |
| P13010 | XRCC5 | 0,014768419 | 0,277912 | X-ray repair cross-complementing protein 5 |
| P60866 | RPS20 | 0,022510574 | 0,283518 | 40S ribosomal protein S20 |
| P45974 | USP5 | 0,003453685 | 0,332138 | Ubiquitin carboxyl-terminal hydrolase 5 |
| Q01518 | CAP1 | 0,021657805 | 0,34699 | Adenylyl cyclase-associated protein 1 |
| Q09028 | RBBP4 | 0,029610025 | 0,348481 | Histone-binding protein RBBP4 |
| P28072 | PSMB6 | 0,037833008 | 0,357829 | Proteasome subunit beta type-6 |
| Q13162 | PRDX4 | 0,015603514 | 0,38355 | Peroxiredoxin-4 |
| P60660 | MYL6 | 0,044835777 | 0,390278 | Myosin light polypeptide 6 |
| P46777 | RPL5 | 0,025315587 | 0,39098 | 60S ribosomal protein L5 |
| Q15233 | NONO | 0,007644441 | 0,394322 | Non-POU domain-containing octamer-binding protein |
| P63167 | DYNLL1 | 0,006185918 | 0,395758 | Dynein light chain 1_ cytoplasmic |
| P42167 | TMPO | 0,030750901 | 0,400491 | Lamina-associated polypeptide 2_ isoforms beta/gamma |
| Q9P0L0 | VAPA | 0,044320982 | 0,406468 | Vesicle-associated membrane protein-associated protein A |
| P23528 | CFL1 | 0,002073821 | 0,414469 | Cofilin-1 |
| P00568 | AK1 | 0,029184665 | 0,428698 | Adenylate kinase isoenzyme 1 |
| O00116 | AGPS | 0,044950831 | 0,431596 | Alkyldihydroxyacetonephosphate synthase_ peroxisomal |
| O00299 | CLIC1 | 0,004360915 | 0,43429 | Chloride intracellular channel protein 1 |
| Q9Y281 | CFL2 | 0,00079957 | 0,43555 | Cofilin-2 |
| P19338 | NCL | 0,000604761 | 0,436393 | Nucleolin |
| P62847 | RPS24 | 0,003957375 | 0,438387 | 40S ribosomal protein S24 |
| P52597 | HNRNPF | 0,041814509 | 0,452499 | Heterogeneous nuclear ribonucleoprotein F |
| Q15019 | SEPT2 | 0,039083798 | 0,459591 | Septin-2 |
| P20674 | COX5A | 0,018121079 | 0,478237 | Cytochrome c oxidase subunit 5A_ mitochondrial |
| P22314 | UBA1 | 0,027916165 | 0,48572 | Ubiquitin-like modifier-activating enzyme 1 |
| P55786 | NPEPPS | 0,01876566 | 0,497831 | Puromycin-sensitive aminopeptidase |
| P46782 | RPS5 | 0,002356033 | 0,50161 | 40S ribosomal protein S5 |
| Q16629 | SRSF7 | 0,000743402 | 0,504793 | Serine/arginine-rich splicing factor 7 |
| P38646 | HSPA9 | 0,012902082 | 0,507003 | Stress-70 protein_ mitochondrial |
| O15523 | DDX3Y | 0,041662645 | 0,508492 | ATP-dependent RNA helicase DDX3Y |
| P62888 | RPL30 | 0,042681499 | 0,517596 | 60S ribosomal protein L30 |
| Q08257 | CRYZ | 0,016030831 | 0,523911 | Quinone oxidoreductase |
| P09471 | GNAO1 | 0,016166271 | 0,534404 | Guanine nucleotide-binding protein G(o) subunit alpha |
| P30040 | ERP29 | 0,035053607 | 0,536244 | Endoplasmic reticulum resident protein 29 |
| P35232 | PHB | 0,038240846 | 0,543398 | Prohibitin |
| P05556 | ITGB1 | 0,037436674 | 0,555399 | Integrin beta-1 |
| Q9Y2Z0 | SUGT1 | 0,000823672 | 0,562228 | Protein SGT1 homolog |
| P26583 | HMGB2 | 0,037495001 | 0,565413 | High mobility group protein B2 |
| P12004 | PCNA | 0,002746673 | 0,568044 | Proliferating cell nuclear antigen |
| Q8IZP2 | ST13P4 | 0,000700785 | 0,568378 | Putative protein FAM10A4 |
| O95757 | HSPA4L | 0,003986809 | 0,569607 | Heat shock 70 kDa protein 4L |
| Q12874 | SF3A3 | 0,011518321 | 0,570035 | Splicing factor 3A subunit 3 |
| Q9UKM9 | RALY | 0,023321408 | 0,581899 | RNA-binding protein Raly |
| P68363 | TUBA1B | 0,025631894 | 0,592792 | Tubulin alpha-1B chain |
| Q9UBT2 | UBA2 | 0,029062246 | 0,59873 | SUMO-activating enzyme subunit 2 |
| P09874 | PARP1 | 7,05031E-07 | 0,59997 | Poly [ADP-ribose] polymerase 1 |
| Q16352 | INA | 0,009242692 | 0,608001 | Alpha-internexin |
| Q8IYT4 | KATNAL2 | 0,031998835 | 0,612949 | Katanin p60 ATPase-containing subunit A-like 2 |
| Q9BRK5 | SDF4 | 0,010091266 | 0,614473 | 45 kDa calcium-binding protein |
| P18669 | PGAM1 | 0,02080662 | 0,627757 | Phosphoglycerate mutase 1 |
| P04899 | GNAI2 | 0,01542734 | 0,630058 | Guanine nucleotide-binding protein G(i) subunit alpha-2 |
| P35244 | RPA3 | 0,045276374 | 0,647558 | Replication protein A 14 kDa subunit |
| P62805 | HIST1H4A | 0,024635044 | 0,647949 | Histone H4 |
| Q9Y295 | DRG1 | 0,005100793 | 0,654502 | Developmentally-regulated GTP-binding protein 1 |
| Q03113 | GNA12 | 0,000995483 | 0,655354 | Guanine nucleotide-binding protein subunit alpha-12 |
| P62807 | HIST1H2BC | 0,007020675 | 0,656595 | Histone H2B type 1-C/E/F/G/I |
| P62906 | RPL10A | 0,011155209 | 0,663428 | 60S ribosomal protein L10a |
| Q9Y5J7 | TIMM9 | 0,020406619 | 0,666289 | Mitochondrial import inner membrane translocase subunit Tim9 |
| P55209 | NAP1L1 | 0,007530095 | 0,66648 | Nucleosome assembly protein 1-like 1 |
| O14737 | PDCD5 | 0,006388572 | 0,667305 | Programmed cell death protein 5 |
| P04908 | HIST1H2AB | 0,029163718 | 0,669795 | Histone H2A type 1-B/E |
| Q15691 | MAPRE1 | 0,041172236 | 0,674297 | Microtubule-associated protein RP/EB family member 1 |
| P27824 | CANX | 0,010642722 | 0,682879 | Calnexin |
| P05386 | RPLP1 | 0,019050146 | 0,683384 | 60S acidic ribosomal protein P1 |
| P07737 | PFN1 | 0,013882718 | 0,685119 | Profilin-1 |
| Q9UI15 | TAGLN3 | 0,003246693 | 0,688464 | Transgelin-3 |
| P34932 | HSPA4 | 0,011748088 | 0,690639 | Heat shock 70 kDa protein 4 |
| Q96DA2 | RAB39B | 0,019016184 | 0,696747 | Ras-related protein Rab-39B |
| P17987 | TCP1 | 0,002133005 | 0,702573 | T-complex protein 1 subunit alpha |
| Q16543 | CDC37 | 0,004948724 | 0,707254 | Hsp90 co-chaperone Cdc37 |
| Q6FI13 | HIST2H2AA3 | 0,016056366 | 0,711122 | Histone H2A type 2-A |
| P04844 | RPN2 | 0,044724924 | 0,711349 | Dolichyl-diphosphooligosaccharide--protein glycosyltransferase subunit 2 |
| P46779 | RPL28 | 0,004770145 | 0,715598 | 60S ribosomal protein L28 |
| P35998 | PSMC2 | 0,010351126 | 0,725035 | 26S proteasome regulatory subunit 7 |
| Q00610 | CLTC | 0,00193601 | 0,727054 | Clathrin heavy chain 1 |
| Q99523 | SORT1 | 0,018712638 | 0,729782 | Sortilin |
| Q92804 | TAF15 | 0,002489739 | 0,733153 | TATA-binding protein-associated factor 2N |
| P23284 | PPIB | 0,001093579 | 0,744399 | Peptidyl-prolyl cis-trans isomerase B |
| P61956 | SUMO2 | 0,003543672 | 0,748098 | Small ubiquitin-related modifier 2 |
| Q9BQG0 | MYBBP1A | 0,019595672 | 0,763224 | Myb-binding protein 1A |
| P0DMV8 | HSPA1A | 0,000537832 | 0,775916 | Heat shock 70 kDa protein 1A |
| Q9NRY5 | FAM114A2 | 0,047722476 | 0,782912 | Protein FAM114A2 |
| P61247 | RPS3A | 0,000362039 | 0,787513 | 40S ribosomal protein S3a |
| P54105 | CLNS1A | 0,042881417 | 0,789996 | Methylosome subunit pICln |
| Q13185 | CBX3 | 2,39975E-05 | 0,793242 | Chromobox protein homolog 3 |
| Q16836 | HADH | 0,014941668 | 0,793921 | Hydroxyacyl-coenzyme A dehydrogenase_ mitochondrial |
| P67809 | YBX1 | 0,015144376 | 0,795667 | Nuclease-sensitive element-binding protein 1 |
| P51991 | HNRNPA3 | 0,001009518 | 0,799712 | Heterogeneous nuclear ribonucleoprotein A3 |
| P00505 | GOT2 | 0,00130553 | 0,80014 | Aspartate aminotransferase_ mitochondrial |
| P30101 | PDIA3 | 0,005582649 | 0,800909 | Protein disulfide-isomerase A3 |
| P13693 | TPT1 | 0,019197756 | 0,801979 | Translationally-controlled tumor protein |
| P27797 | CALR | 0,016169348 | 0,805385 | Calreticulin |
| Q07021 | C1QBP | 0,021928922 | 0,81521 | Complement component 1 Q subcomponent-binding protein_ mitochondrial |
| P68032 | ACTC1 | 0,036492916 | 0,817339 | Actin_ alpha cardiac muscle 1 |
| Q8NHW5 | RPLP0P6 | 0,044049063 | 0,817644 | 60S acidic ribosomal protein P0-like |
| Q9C0A0 | CNTNAP4 | 0,018782167 | 0,823072 | Contactin-associated protein-like 4 |
| O00429 | DNM1L | 0,037264939 | 0,825961 | Dynamin-1-like protein |
| P61204 | ARF3 | 0,000527816 | 0,834318 | ADP-ribosylation factor 3 |
| Q7Z4H8 | KDELC2 | 0,014846678 | 0,839293 | KDEL motif-containing protein 2 |
| P68402 | PAFAH1B2 | 0,018849861 | 0,850628 | Platelet-activating factor acetylhydrolase IB subunit beta |
| Q14498 | RBM39 | 0,008955218 | 0,85132 | RNA-binding protein 39 |
| P09211 | GSTP1 | 0,000877149 | 0,856982 | Glutathione S-transferase P |
| P32969 | RPL9 | 0,000138147 | 0,859797 | 60S ribosomal protein L9 |
| P0DN76 | U2AF1L5 | 0,015600938 | 0,873256 | Splicing factor U2AF 35 kDa subunit-like protein |
| Q15084 | PDIA6 | 0,012979433 | 0,884933 | Protein disulfide-isomerase A6 |
| P00367 | GLUD1 | 0,016009407 | 0,88982 | Glutamate dehydrogenase 1_ mitochondrial |
| P06744 | GPI | 0,004570019 | 0,891527 | Glucose-6-phosphate isomerase |
| Q15029 | EFTUD2 | 0,031045754 | 0,892221 | 116 kDa U5 small nuclear ribonucleoprotein component |
| Q9UKD2 | MRTO4 | 0,005911844 | 0,902712 | mRNA turnover protein 4 homolog |
| Q9BQ39 | DDX50 | 0,002466031 | 0,905347 | ATP-dependent RNA helicase DDX50 |
| Q56UQ5 | #VALOR! | 0,020125326 | 0,907275 | TPT1-like protein |
| P08185 | SERPINA6 | 0,031821977 | 0,91289 | Corticosteroid-binding globulin |
| Q9Y605 | MRFAP1 | 0,040156933 | 0,923885 | MORF4 family-associated protein 1 |
| P61026 | RAB10 | 0,003793946 | 0,935536 | Ras-related protein Rab-10 |
| Q99623 | PHB2 | 0,00126046 | 0,936684 | Prohibitin-2 |
| P48681 | NES | 0,02492726 | 0,939852 | Nestin |
| O96019 | ACTL6A | 0,04397344 | 0,941007 | Actin-like protein 6A |
| P30153 | PPP2R1A | 0,012987423 | 0,948118 | Serine/threonine-protein phosphatase 2A 65 kDa regulatory subunit A alpha isoform |
| P39656 | DDOST | 0,007841138 | 0,949547 | Dolichyl-diphosphooligosaccharide--protein glycosyltransferase 48 kDa subunit |
| Q9H3N1 | TMX1 | 1,075E-05 | 0,954644 | Thioredoxin-related transmembrane protein 1 |
| O15247 | CLIC2 | 0,031982914 | 0,958059 | Chloride intracellular channel protein 2 |
| Q9P1U1 | ACTR3B | 0,026920629 | 0,962325 | Actin-related protein 3B |
| P62277 | RPS13 | 0,000999239 | 0,964161 | 40S ribosomal protein S13 |
| P49588 | AARS | 0,000219304 | 0,97009 | Alanine--tRNA ligase_ cytoplasmic |
| P26599 | PTBP1 | 0,00105753 | 0,975819 | Polypyrimidine tract-binding protein 1 |
| A1L0T0 | ILVBL | 0,014433459 | 0,982688 | Acetolactate synthase-like protein |
| Q8N0Y7 | PGAM4 | 0,00036351 | 0,98492 | Probable phosphoglycerate mutase 4 |
| Q9BYZ2 | LDHAL6B | 0,006753155 | 0,991901 | L-lactate dehydrogenase A-like 6B |
| Q9GZV4 | EIF5A2 | 0,005683888 | 0,996676 | Eukaryotic translation initiation factor 5A-2 |
| Q9NRW1 | RAB6B | 0,000184202 | 0,997608 | Ras-related protein Rab-6B |
| Q8WVV9 | HNRNPLL | 0,000361162 | 1,002388 | Heterogeneous nuclear ribonucleoprotein L-like |
| P49321 | NASP | 3,60346E-07 | 1,004125 | Nuclear autoantigenic sperm protein |
| P35613 | BSG | 0,047851919 | 1,011413 | Basigin |
| P02790 | HPX | 0,03747854 | 1,020742 | Hemopexin |
| Q9NQA5 | TRPV5 | 0,038903022 | 1,033718 | Transient receptor potential cation channel subfamily V member 5 |
| P52815 | MRPL12 | 0,036578394 | 1,050934 | 39S ribosomal protein L12_ mitochondrial |
| Q05639 | EEF1A2 | 0,014802075 | 1,062581 | Elongation factor 1-alpha 2 |
| P68400 | CSNK2A1 | 0,036785689 | 1,064523 | Casein kinase II subunit alpha |
| P62318 | SNRPD3 | 0,024804434 | 1,068029 | Small nuclear ribonucleoprotein Sm D3 |
| P23497 | SP100 | 0,006247361 | 1,073584 | Nuclear autoantigen Sp-100 |
| Q14103 | HNRNPD | 0,000116955 | 1,076687 | Heterogeneous nuclear ribonucleoprotein D0 |
| P21291 | CSRP1 | 0,026703666 | 1,078126 | Cysteine and glycine-rich protein 1 |
| P62314 | SNRPD1 | 0,009746294 | 1,09074 | Small nuclear ribonucleoprotein Sm D1 |
| P31040 | SDHA | 0,016931726 | 1,110436 | Succinate dehydrogenase [ubiquinone] flavoprotein subunit_ mitochondrial |
| P63244 | RACK1 | 0,006237711 | 1,116766 | Receptor of activated protein C kinase 1 |
| P04083 | ANXA1 | 0,007297984 | 1,12132 | Annexin A1 |
| P99999 | CYCS | 0,015533468 | 1,124734 | Cytochrome c |
| Q9NWB7 | IFT57 | 0,023529315 | 1,130677 | Intraflagellar transport protein 57 homolog |
| P02008 | HBZ | 0,021714089 | 1,139451 | Hemoglobin subunit zeta |
| P78527 | PRKDC | 3,37899E-05 | 1,151244 | DNA-dependent protein kinase catalytic subunit |
| P63241 | EIF5A | 0,002094699 | 1,155413 | Eukaryotic translation initiation factor 5A-1 |
| P08133 | ANXA6 | 0,004513906 | 1,172772 | Annexin A6 |
| Q01130 | SRSF2 | 1,33743E-05 | 1,179425 | Serine/arginine-rich splicing factor 2 |
| P61923 | COPZ1 | 0,002042977 | 1,216019 | Coatomer subunit zeta-1 |
| A0A0B4J2A2 | PPIAL4C | 0,000136873 | 1,221277 | Peptidyl-prolyl cis-trans isomerase A-like 4C |
| O14929 | HAT1 | 4,29411E-06 | 1,228103 | Histone acetyltransferase type B catalytic subunit |
| Q13561 | DCTN2 | 0,000731508 | 1,238479 | Dynactin subunit 2 |
| P08138 | NGFR | 0,008702447 | 1,249526 | Tumor necrosis factor receptor superfamily member 16 |
| O95716 | RAB3D | 8,92133E-05 | 1,257184 | Ras-related protein Rab-3D |
| Q9HDC9 | APMAP | 0,000541477 | 1,302563 | Adipocyte plasma membrane-associated protein |
| O43237 | DYNC1LI2 | 0,021106506 | 1,314117 | Cytoplasmic dynein 1 light intermediate chain 2 |
| O00499 | BIN1 | 0,047685962 | 1,321112 | Myc box-dependent-interacting protein 1 |
| P62136 | PPP1CA | 0,014464283 | 1,321225 | Serine/threonine-protein phosphatase PP1-alpha catalytic subunit |
| P55036 | PSMD4 | 0,000628033 | 1,33931 | 26S proteasome non-ATPase regulatory subunit 4 |
| P24752 | ACAT1 | 0,000585393 | 1,345 | Acetyl-CoA acetyltransferase_ mitochondrial |
| P0DN79 | CBSL | 0,014155189 | 1,348643 | Cystathionine beta-synthase-like protein |
| Q14344 | GNA13 | 0,004954355 | 1,366547 | Guanine nucleotide-binding protein subunit alpha-13 |
| P60709 | ACTB | 9,48547E-05 | 1,367462 | Actin_ cytoplasmic 1 |
| P20340 | RAB6A | 2,04573E-05 | 1,372752 | Ras-related protein Rab-6A |
| Q58FF3 | HSP90B2P | 0,005004422 | 1,38498 | Putative endoplasmin-like protein |
| P09622 | DLD | 0,003023899 | 1,399329 | Dihydrolipoyl dehydrogenase_ mitochondrial |
| Q9NQC3 | RTN4 | 0,005988447 | 1,426101 | Reticulon-4 |
| P35637 | FUS | 0,000147086 | 1,432229 | RNA-binding protein FUS |
| Q9Y224 | RTRAF | 0,00022517 | 1,438748 | RNA transcription_ translation and transport factor protein |
| Q9H0A0 | NAT10 | 0,001538655 | 1,492657 | RNA cytidine acetyltransferase |
| Q9NS69 | TOMM22 | 0,011107013 | 1,505403 | Mitochondrial import receptor subunit TOM22 homolog |
| Q9UNH7 | SNX6 | 0,001516745 | 1,514001 | Sorting nexin-6 |
| Q8WVJ2 | NUDCD2 | 0,034576234 | 1,531245 | NudC domain-containing protein 2 |
| Q8IUE6 | HIST2H2AB | 0,007798856 | 1,532516 | Histone H2A type 2-B |
| Q9BPU6 | DPYSL5 | 0,002151233 | 1,536203 | Dihydropyrimidinase-related protein 5 |
| P00403 | MT-CO2 | 0,008037149 | 1,558534 | Cytochrome c oxidase subunit 2 |
| P59190 | RAB15 | 0,002767549 | 1,587861 | Ras-related protein Rab-15 |
| Q6PEY2 | TUBA3E | 0,01073907 | 1,602647 | Tubulin alpha-3E chain |
| P16615 | ATP2A2 | 9,40245E-06 | 1,616134 | Sarcoplasmic/endoplasmic reticulum calcium ATPase 2 |
| Q9NVE7 | PANK4 | 0,00014778 | 1,650055 | Pantothenate kinase 4 |
| P29966 | MARCKS | 0,001770479 | 1,661932 | Myristoylated alanine-rich C-kinase substrate |
| P06733 | ENO1 | 6,26384E-05 | 1,666898 | Alpha-enolase |
| Q9NY12 | GAR1 | 0,000255294 | 1,677638 | H/ACA ribonucleoprotein complex subunit 1 |
| Q9BWJ5 | SF3B5 | 0,009316848 | 1,684436 | Splicing factor 3B subunit 5 |
| Q9Y6M1 | IGF2BP2 | 9,90664E-05 | 1,687951 | Insulin-like growth factor 2 mRNA-binding protein 2 |
| O00232 | PSMD12 | 0,000375475 | 1,705939 | 26S proteasome non-ATPase regulatory subunit 12 |
| O43169 | CYB5B | 0,005031842 | 1,731126 | Cytochrome b5 type B |
| P05387 | RPLP2 | 0,000199962 | 1,75622 | 60S acidic ribosomal protein P2 |
| Q9NY65 | TUBA8 | 0,000118418 | 1,765552 | Tubulin alpha-8 chain |
| P14678 | SNRPB | 0,002495411 | 1,791647 | Small nuclear ribonucleoprotein-associated proteins B and B' |
| Q16630 | CPSF6 | 0,007874974 | 1,795527 | Cleavage and polyadenylation specificity factor subunit 6 |
| P32119 | PRDX2 | 0,00284343 | 1,808953 | Peroxiredoxin-2 |
| O60841 | EIF5B | 3,7176E-05 | 1,8374 | Eukaryotic translation initiation factor 5B |
| O14950 | MYL12B | 0,00019407 | 2,001865 | Myosin regulatory light chain 12B |
| Q58FF6 | HSP90AB4P | 0,004772657 | 2,027068 | Putative heat shock protein HSP 90-beta 4 |
| P58546 | MTPN | 0,004088342 | 2,206413 | Myotrophin |
| P53618 | COPB1 | 0,000900501 | 2,211485 | Coatomer subunit beta |
| P56134 | ATP5J2 | 1,22394E-05 | 2,314404 | ATP synthase subunit f_ mitochondrial |
| A6NHL2 | TUBAL3 | 2,95285E-05 | 2,322951 | Tubulin alpha chain-like 3 |
| Q16891 | IMMT | 5,82077E-05 | 2,335733 | MICOS complex subunit MIC60 |
| P61619 | SEC61A1 | 0,000559067 | 2,343291 | Protein transport protein Sec61 subunit alpha isoform 1 |
| Q9UMX0 | UBQLN1 | 0,01083007 | 3,202046 | Ubiquilin-1 |
| P20337 | RAB3B | 0,006681361 | 3,232304 | Ras-related protein Rab-3B |
| Q96QV6 | HIST1H2AA | 0,013136159 | 3,237238 | Histone H2A type 1-A |
| Q9NP86 | CABP5 | 7,76465E-05 | 3,701706 | Calcium-binding protein 5 |
| Q92928 | RAB1C | 0,001362399 | 4,062148 | Putative Ras-related protein Rab-1C |
| Q08945 | SSRP1 | 2,23444E-05 | 5,875745 | FACT complex subunit SSRP1 |
