# Supplementary material for: Biochemical Pathways Triggered by Antipsychotics in Human Oligodendrocytes: Potential of Discovering New Treatment Targets
Source: Front Pharmacol. 2019 Mar 5;10:186. doi: 10.3389/fphar.2019.00186 (PMC6411851; doi:10.3389/fphar.2019.00186)
Supplement: Table S3 — Ingenuity canonical pathway analysis for oligodendrocyte treated with first generation antipsychotics. [file Table_3.DOCX]

| **Table 3 - Common and unique canonical pathways in analysis of oligodendrocyte treated with chlorpromazine or haloperidol** | | | | | | | |
| --- | --- | --- | --- | --- | --- | --- | --- |
| Treatment | Ingenuity Canonical Pathways | p-value | Ratio | Treatment | Ingenuity Canonical Pathways | p-value | Ratio |
| Chlorpromazine | EIF2 Signaling | 3,98107E-17 | 22/221 (0,0995) | Haloperidol | EIF2 Signaling | 6,30957E-15 | 24/221 (0,109) |
| Chlorpromazine | Regulation of eIF4 and p70S6K Signaling | 1,41254E-07 | 11/157 (0,0701) | Haloperidol | Remodeling of Epithelial Adherens Junctions | 2,5704E-09 | 11/69 (0,159) |
| Chlorpromazine | Protein Ubiquitination Pathway | 4,0738E-06 | 12/265 (0,0453) | Haloperidol | Ephrin B Signaling | 4,7863E-09 | 11/73 (0,151) |
| Chlorpromazine | Unfolded protein response | 8,51138E-06 | 6/55 (0,109) | Haloperidol | Signaling by Rho Family GTPases | 1,54882E-08 | 18/252 (0,0714) |
| Chlorpromazine | mTOR Signaling | 1,14815E-05 | 10/201 (0,0498) | Haloperidol | RhoA Signaling | 1,77828E-08 | 13/124 (0,105) |
| Chlorpromazine | RhoA Signaling | 1,38038E-05 | 8/124 (0,0645) | Haloperidol | Germ Cell-Sertoli Cell Junction Signaling | 1,90546E-08 | 15/173 (0,0867) |
| Chlorpromazine | Pyrimidine Deoxyribonucleotides De Novo Biosynthesis I | 4,36516E-05 | 4/23 (0,174) | Haloperidol | Axonal Guidance Signaling | 2,23872E-08 | 24/453 (0,053) |
| Chlorpromazine | Glycolysis I | 7,24436E-05 | 4/26 (0,154) | Haloperidol | RhoGDI Signaling | 2,63027E-08 | 13/124 (0,0847) |
| Chlorpromazine | Cell Cycle: G2/M DNA Damage Checkpoint Regulation | 7,4131E-05 | 5/50 (0,1) | Haloperidol | Protein Ubiquitination Pathway | 3,38844E-08 | 18/265 (0,0679) |
| Chlorpromazine | Aldosterone Signaling in Epithelial Cells | 0,000120226 | 8/168 (0,0476) | Haloperidol | Regulation of Actin-based Motility by Rho | 4,57088E-08 | 11/90 (0,122) |
| Chlorpromazine | Mitochondrial Dysfunction | 0,000134896 | 8/171 (0,0468) | Haloperidol | Unfolded protein response | 5,88844E-08 | 9/55 (0,164) |
| Chlorpromazine | Endoplasmic Reticulum Stress Pathway | 0,000776247 | 3/21 (0,143) | Haloperidol | Actin Cytoskeleton Signaling | 6,76083E-07 | 15/227 (0,0661) |
| Chlorpromazine | PI3K/AKT Signaling | 0,000812831 | 6/125 (0,048) | Haloperidol | Ephrin Receptor Signaling | 1,02329E-06 | 13/175 (0,0743) |
| Chlorpromazine | Actin Cytoskeleton Signaling | 0,000891251 | 8/227 (0,0352) | Haloperidol | Phagosome Maturation | 1,04713E-06 | 12/148 (0,0811) |
| Chlorpromazine | Gluconeogenesis I | 0,001479108 | 3/26 (0,115) | Haloperidol | Breast Cancer Regulation by Stathmin1 | 1,07152E-06 | 14/205 (0,0683) |
| Chlorpromazine | Superpathway of Serine and Glycine Biosynthesis I | 0,001548817 | 2/7 (0,286) | Haloperidol | Epithelial Adherens Junction Signaling | 6,0256E-06 | 11/146 (0,0753) |
| Chlorpromazine | Salvage Pathways of Pyrimidine Ribonucleotides | 0,00162181 | 5/97 (0,0515) | Haloperidol | Semaphorin Signaling in Neurons | 7,94328E-06 | 7/53 (0,132) |
| Chlorpromazine | Signaling by Rho Family GTPases | 0,001737801 | 8/252 (0,0317) | Haloperidol | Regulation of eIF4 and p70S6K Signaling | 1,20226E-05 | 11/157 (0,0701) |
| Chlorpromazine | IGF-1 Signaling | 0,002398833 | 5/106 (0,0472) | Haloperidol | ILK Signaling | 2,04174E-05 | 12/197 (0,0609) |
| Chlorpromazine | Folate Transformations I | 0,002630268 | 2/9 (0,222) | Haloperidol | Tec Kinase Signaling | 2,5704E-05 | 11/170 (0,0647) |
| Chlorpromazine | ERK5 Signaling | 0,002691535 | 4/66 (0,0606) | Haloperidol | Granzyme B Signaling | 5,62341E-05 | 4/16 (0,25) |
| Chlorpromazine | Myc Mediated Apoptosis Signaling | 0,003311311 | 4/70 (0,0571) | Haloperidol | Caveolar-mediated Endocytosis Signaling | 5,62341E-05 | 7/71 (0,0986) |
| Chlorpromazine | eNOS Signaling | 0,004073803 | 6/172 (0,0349) | Haloperidol | CXCR4 Signaling | 0,000102329 | 10/165 (0,0606) |
| Chlorpromazine | RhoGDI Signaling | 0,004786301 | 6/177 (0,0339) | Haloperidol | mTOR Signaling | 0,00011749 | 11/201 (0,0547) |
| Chlorpromazine | 14-3-3-mediated Signaling | 0,006025596 | 5/131 (0,0382) | Haloperidol | Sertoli Cell-Sertoli Cell Junction Signaling | 0,000190546 | 10/178 (0,0562) |
| Chlorpromazine | p70S6K Signaling | 0,00616595 | 5/132 (0,0379) | Haloperidol | Spliceosomal Cycle | 0,000190546 | 2/2 (1) |
| Chlorpromazine | Axonal Guidance Signaling | 0,00676083 | 10/453 (0,0221) | Haloperidol | Sirtuin Signaling Pathway | 0,000229087 | 13/292 (0,0445) |
| Chlorpromazine | Pyrimidine Ribonucleotides Interconversion | 0,007079458 | 3/45 (0,0667) | Haloperidol | Sumoylation Pathway | 0,000371535 | 7/96 (0,0729) |
| Chlorpromazine | Neuregulin Signaling | 0,007413102 | 4/88 (0,0455) | Haloperidol | Gap Junction Signaling | 0,000398107 | 10/195 (0,0513) |
| Chlorpromazine | Pyrimidine Ribonucleotides De Novo Biosynthesis | 0,008128305 | 3/47 (0,0638) | Haloperidol | Glycolysis I | 0,000416869 | 4/26 (0,154) |
| Chlorpromazine | Aryl Hydrocarbon Receptor Signaling | 0,008128305 | 5/141 (0,0355) | Haloperidol | Gluconeogenesis I | 0,000416869 | 4/26 (0,154) |
| Chlorpromazine | ERK/MAPK Signaling | 0,00851138 | 6/200 (0,03) | Haloperidol | 14-3-3-mediated Signaling | 0,00047863 | 8/131 (0,0611) |
| Chlorpromazine | Epithelial Adherens Junction Signaling | 0,009332543 | 5/146 (0,0342) | Haloperidol | Tight Junction Signaling | 0,000537032 | 9/167 (0,0539) |
| Chlorpromazine | RAN Signaling | 0,009549926 | 2/17 (0,118) | Haloperidol | Thrombin Signaling | 0,000562341 | 10/204 (0,049) |
| Chlorpromazine | Prostate Cancer Signaling | 0,010471285 | 4/97 (0,0412) | Haloperidol | Huntington's Disease Signaling | 0,000758578 | 11/250 (0,044) |
| Chlorpromazine | PAK Signaling | 0,012022644 | 4/101 (0,0396) | Haloperidol | DNA Double-Strand Break Repair by Non-Homologous End Joining | 0,000851138 | 3/14 (0,214) |
| Chlorpromazine | Granzyme A Signaling | 0,013182567 | 2/20 (0,1) | Haloperidol | Integrin Signaling | 0,000977237 | 10/219 (0,0457) |
| Chlorpromazine | Regulation of Cellular Mechanics by Calpain Protease | 0,013803843 | 3/57 (0,0526) | Haloperidol | Cardiac Hypertrophy Signaling | 0,001659587 | 10/235 (0,0426) |
| Chlorpromazine | Choline Degradation I | 0,017378008 | 1/2 (0,5) | Haloperidol | Role of Tissue Factor in Cancer | 0,001698244 | 7/124 (0,0565) |
| Chlorpromazine | Glycine Biosynthesis I | 0,017378008 | 1/2 (0,5) | Haloperidol | IL-8 Signaling | 0,001737801 | 9/197 (0,0457) |
| Chlorpromazine | Germ Cell-Sertoli Cell Junction Signaling | 0,018197009 | 5/173 (0,0289) | Haloperidol | Antigen Presentation Pathway | 0,001819701 | 4/38 (0,105) |
| Chlorpromazine | Ephrin Receptor Signaling | 0,019054607 | 5/175 (0,0286) | Haloperidol | Mechanisms of Viral Exit from Host Cells | 0,002398833 | 4/41 (0,0976) |
| Chlorpromazine | Tryptophan Degradation X (Mammalian, via Tryptamine) | 0,019952623 | 2/25 (0,08) | Haloperidol | Clathrin-mediated Endocytosis Signaling | 0,002398833 | 9/207 (0,0435) |
| Chlorpromazine | Mitotic Roles of Polo-Like Kinase | 0,019952623 | 3/66 (0,0455) | Haloperidol | Agrin Interactions at Neuromuscular Junction | 0,002630268 | 5/69 (0,0725) |
| Chlorpromazine | Sertoli Cell-Sertoli Cell Junction Signaling | 0,020417379 | 5/178 (0,0281) | Haloperidol | Pyruvate Fermentation to Lactate | 0,002754229 | 2/6 (0,333) |
| Chlorpromazine | Remodeling of Epithelial Adherens Junctions | 0,022908677 | 3/69 (0,0435) | Haloperidol | PAK Signaling | 0,002818383 | 6/101 (0,0594) |
| Chlorpromazine | Huntington's Disease Signaling | 0,022908677 | 6/250 (0,024) | Haloperidol | Androgen Signaling | 0,002951209 | 7/137 (0,0511) |
| Chlorpromazine | 5-aminoimidazole Ribonucleotide Biosynthesis I | 0,02630268 | 1/3 (0,333) | Haloperidol | Molecular Mechanisms of Cancer | 0,003467369 | 13/394 (0,033) |
| Chlorpromazine | D-glucuronate Degradation I | 0,02630268 | 1/3 (0,333) | Haloperidol | Protein Kinase A Signaling | 0,003981072 | 13/401 (0,0324) |
| Chlorpromazine | Oxidized GTP and dGTP Detoxification | 0,02630268 | 1/3 (0,333) | Haloperidol | Oxidative Phosphorylation | 0,004073803 | 6/109 (0,055) |
| Chlorpromazine | Ephrin B Signaling | 0,02630268 | 3/73 (0,0411) | Haloperidol | TCA Cycle II (Eukaryotic) | 0,004265795 | 3/24 (0,125) |
| Chlorpromazine | IL-6 Signaling | 0,02630268 | 4/128 (0,0312) | Haloperidol | Virus Entry via Endocytic Pathways | 0,004265795 | 6/110 (0,0545) |
| Chlorpromazine | RAR Activation | 0,02630268 | 5/190 (0,0263) | Haloperidol | Lipid Antigen Presentation by CD1 | 0,005370318 | 3/21 (0,115) |
| Chlorpromazine | Hypoxia Signaling in the Cardiovascular System | 0,028183829 | 3/75 (0,04) | Haloperidol | Actin Nucleation by ARP-WASP Complex | 0,007413102 | 4/56 (0,0714) |
| Chlorpromazine | ILK Signaling | 0,029512092 | 5/197 (0,0254) | Haloperidol | Calcium Transport I | 0,007943282 | 2/10 (0,2) |
| Chlorpromazine | Non-Small Cell Lung Cancer Signaling | 0,030199517 | 3/77 (0,039) | Haloperidol | Sphingosine-1-phosphate Signaling | 0,007943282 | 6/125 (0,048) |
| Chlorpromazine | Glucocorticoid Receptor Signaling | 0,030199517 | 7/339 (0,0206) | Haloperidol | IL-1 Signaling | 0,008912509 | 5/92 (0,0543) |
| Chlorpromazine | VDR/RXR Activation | 0,030902954 | 3/78 (0,0385) | Haloperidol | Aldosterone Signaling in Epithelial Cells | 0,008912509 | 7/168 (0,0417) |
| Chlorpromazine | Gα12/13 Signaling | 0,030902954 | 4/135 (0,0296) | Haloperidol | Death Receptor Signaling | 0,009332543 | 5/93 (0,0538) |
| Chlorpromazine | Cyclins and Cell Cycle Regulation | 0,033884416 | 3/81 (0,037) | Haloperidol | Ephrin A Signaling | 0,009549926 | 4/60 (0,0667) |
| Chlorpromazine | Pentose Phosphate Pathway (Oxidative Branch) | 0,034673685 | 1/4 (0,25) | Haloperidol | CCR3 Signaling in Eosinophils | 0,009549926 | 6/130 (0,0462) |
| Chlorpromazine | HIPPO signaling | 0,040738028 | 3/87 (0,0345) | Haloperidol | Cellular Effects of Sildenafil (Viagra) | 0,009772372 | 6/131 (0,0458) |
| Chlorpromazine | Ethanol Degradation II | 0,041686938 | 2/37 (0,0541) | Haloperidol | Synaptic Long Term Depression | 0,010715193 | 7/174 ()0,0402 |
| Chlorpromazine | Bladder Cancer Signaling | 0,041686938 | 3/88 (0,0341) | Haloperidol | BER pathway | 0,011481536 | 2/12 (0,167) |
| Chlorpromazine | Phagosome Maturation | 0,041686938 | 4/148 (0,027) | Haloperidol | CDK5 Signaling | 0,012022644 | 5/99 (0,0505) |
| Chlorpromazine | Creatine-phosphate Biosynthesis | 0,042657952 | 1/5 (0,2) | Haloperidol | Superpathway of Methionine Degradation | 0,014454398 | 3/37 (0,0811) |
| Chlorpromazine | Tetrahydrofolate Salvage from 5,10-methenyltetrahydrofolate | 0,042657952 | 1/5 (0,2) | Haloperidol | Isoleucine Degradation I | 0,015488166 | 2/14 (0,143) |
| Chlorpromazine | Serine Biosynthesis | 0,042657952 | 1/5 (0,2) | Haloperidol | Telomere Extension by Telomerase | 0,017782794 | 2/15 (0,133) |
| Chlorpromazine | Lysine Degradation II | 0,042657952 | 1/5 (0,2) | Haloperidol | Phospholipase C Signaling | 0,019498446 | 8/242 (0,0331) |
| Chlorpromazine | Lysine Degradation V | 0,042657952 | 1/5 (0,2) | Haloperidol | Glucocorticoid Receptor Signaling | 0,019952623 | 10/339 ()0,0295 |
| Chlorpromazine | dTMP De Novo Biosynthesis | 0,042657952 | 1/5 (0,2) | Haloperidol | Glutaryl-CoA Degradation | 0,019952623 | 2/16 (0,125) |
| Chlorpromazine | Folate Polyglutamylation | 0,042657952 | 1/5 (0,2) | Haloperidol | G Beta Gamma Signaling | 0,020892961 | 5/114 (0,0439) |
| Chlorpromazine | Sirtuin Signaling Pathway | 0,043651583 | 6/292 (0,0205) | Haloperidol | Rac Signaling | 0,023442288 | 5/117 (0,0427) |
| Chlorpromazine | Regulation of Actin-based Motility by Rho | 0,044668359 | 3/90 (0,0333) | Haloperidol | Dopamine-DARPP32 Feedback in cAMP Signaling | 0,02630268 | 6/164 (0,0366) |
| Chlorpromazine | Apoptosis Signaling | 0,044668359 | 3/90 (0,0333) | Haloperidol | Palmitate Biosynthesis I (Animals) | 0,027542287 | 1/2 (0,5) |
| Chlorpromazine | tRNA Charging | 0,045708819 | 2/39 (0,0513) | Haloperidol | Fatty Acid Biosynthesis Initiation II | 0,027542287 | 1/2 (0,5) |
| Chlorpromazine | Noradrenaline and Adrenaline Degradation | 0,047863009 | 2/40 (0,05) | Haloperidol | Cysteine Biosynthesis/Homocysteine Degradation | 0,027542287 | 1/2 (0,5) |
| Shared between chlorpromazine and haloperidol  Specific to haloperidol  Specific to chlorpromazine | | | | Haloperidol | Glutamate Biosynthesis II | 0,027542287 | 1/2 (0,5) |
|  |  |  |  | Haloperidol | Glutamate Degradation X | 0,027542287 | 1/2 (0,5) |
|  |  |  |  | Haloperidol | GADD45 Signaling | 0,027542287 | 2/19 (0,105) |
|  |  |  |  | Haloperidol | Leukocyte Extravasation Signaling | 0,027542287 | 7/211 (0,0332) |
|  |  |  |  | Haloperidol | Cdc42 Signaling | 0,028840315 | 6/167 (0,0359) |
|  |  |  |  | Haloperidol | Granzyme A Signaling | 0,030902954 | 2/20 (0,1) |
|  |  |  |  | Haloperidol | Cell Cycle: G2/M DNA Damage Checkpoint Regulation | 0,031622777 | 3/50 (0,06) |
|  |  |  |  | Haloperidol | Mitochondrial Dysfunction | 0,031622777 | 6/171 (0,0351) |
|  |  |  |  | Haloperidol | HIPPO signaling | 0,032359366 | 4/87 (0,046) |
|  |  |  |  | Haloperidol | Endoplasmic Reticulum Stress Pathway | 0,033884416 | 2/21 (0,0952) |
|  |  |  |  | Haloperidol | Phagosome Formation | 0,035481339 | 5/131 (0,0382) |
|  |  |  |  | Haloperidol | Apoptosis Signaling | 0,036307805 | 4/90 (0,0444) |
|  |  |  |  | Haloperidol | HMGB1 Signaling | 0,037153523 | 5/133 (0,0376) |
|  |  |  |  | Haloperidol | Gα12/13 Signaling | 0,039810717 | 5/135 (0,037) |
|  |  |  |  | Haloperidol | D-glucuronate Degradation I | 0,040738028 | 1/3 (0,333) |
|  |  |  |  | Haloperidol | Hypusine Biosynthesis | 0,040738028 | 1/3 (0,333) |
|  |  |  |  | Haloperidol | 4-hydroxyproline Degradation I | 0,040738028 | 1/3 (0,333) |
|  |  |  |  | Haloperidol | Glutamate Degradation II | 0,040738028 | 1/3 (0,333) |
|  |  |  |  | Haloperidol | Aspartate Biosynthesis | 0,040738028 | 1/3 (0,333) |
|  |  |  |  | Haloperidol | Glutathione Redox Reactions I | 0,042657952 | 2/24 (0,0833) |
|  |  |  |  | Haloperidol | Cell Cycle Control of Chromosomal Replication | 0,042657952 | 3/56 (0,0536) |
|  |  |  |  | Haloperidol | Role of CHK Proteins in Cell Cycle Checkpoint Control | 0,043651583 | 3/57 (0,0526) |
|  |  |  |  | Haloperidol | Cardiac β-adrenergic Signaling | 0,045708819 | 5/141 (0,0355) |
|  |  |  |  | Haloperidol | Tryptophan Degradation III (Eukaryotic) | 0,046773514 | 2/25 (0,08) |
|  |  |  |  | Haloperidol | FAK Signaling | 0,048977882 | 4/99 (0,0404) |
|  |  |  |  | Haloperidol | D-myo-inositol (1,4,5,6)-Tetrakisphosphate Biosynthesis | 0,048977882 | 5/143 (0,035) |
|  |  |  |  | Haloperidol | D-myo-inositol (3,4,5,6)-tetrakisphosphate Biosynthesis | 0,048977882 | 5/143 (0,035) |
| Haloperidol | Endothelin-1 Signaling | 0,048977882 | 6/190 (0,0316) |  |  |  |  |
