# Supplementary material for: Biochemical Pathways Triggered by Antipsychotics in Human Oligodendrocytes: Potential of Discovering New Treatment Targets
Source: Front Pharmacol. 2019 Mar 5;10:186. doi: 10.3389/fphar.2019.00186 (PMC6411851; doi:10.3389/fphar.2019.00186)
Supplement: Table S4 — Proteins affected by quetiapine treatment. [file Table_4.DOCX]

| Table 4: Proteins affected by quetiapine treatment | | | | |
| --- | --- | --- | --- | --- |
| *Accession* | *Gene* | *Anova (p)* | *Log2 FC* | *Protein* |
| Q96HE7 | ERO1A | 0,035207 | -4,56697 | ERO1-like protein alpha |
| P51665 | PSMD7 | 0,003345 | -2,49739 | 26S proteasome non-ATPase regulatory subunit 7 |
| P82909 | MRPS36 | 0,033394 | -2,20984 | 28S ribosomal protein S36_ mitochondrial |
| Q6P2Q9 | PRPF8 | 0,048574 | 0,803315 | Pre-mRNA-processing-splicing factor 8 |
| Q12765 | SCRN1 | 0,041737 | 1,02017 | Secernin-1 |
| Q92879 | CELF1 | 0,032083 | 1,102601 | CUGBP Elav-like family member 1 |
| Q7Z333 | SETX | 0,037958 | 1,133575 | Probable helicase senataxin |
| Q9BUQ8 | DDX23 | 0,048687 | 1,136942 | Probable ATP-dependent RNA helicase DDX23 |
| P27348 | YWHAQ | 0,034428 | 1,174033 | 14-3-3 protein theta |
| Q9NQ50 | MRPL40 | 0,038745 | 1,40177 | 39S ribosomal protein L40_ mitochondrial |
| Q9Y333 | LSM2 | 0,049292 | 1,474858 | U6 snRNA-associated Sm-like protein LSm2 |
| Q8NE71 | ABCF1 | 0,014376 | 1,493906 | ATP-binding cassette sub-family F member 1 |
| P19387 | POLR2C | 0,030096 | 1,564559 | DNA-directed RNA polymerase II subunit RPB3 |
| Q9UKM7 | MAN1B1 | 0,046105 | 1,697943 | Endoplasmic reticulum mannosyl-oligosaccharide 1_2-alpha-mannosidase |
| Q14257 | RCN2 | 0,043475 | 1,728372 | Reticulocalbin-2 |
| Q7Z3B4 | NUP54 | 0,009327 | 1,868906 | Nucleoporin p54 |
| Q9UBE0 | SAE1 | 0,009665 | 2,540591 | SUMO-activating enzyme subunit 1 |
| P99999 | CYCS | 0,025174 | 2,714411 | Cytochrome c |
| Q13243 | SRSF5 | 0,014395 | Infinity | Serine/arginine-rich splicing factor 5 |
