# Supplementary material for: Biochemical Pathways Triggered by Antipsychotics in Human Oligodendrocytes: Potential of Discovering New Treatment Targets
Source: Front Pharmacol. 2019 Mar 5;10:186. doi: 10.3389/fphar.2019.00186 (PMC6411851; doi:10.3389/fphar.2019.00186)
Supplement: Table S5 — Proteins affected by risperidone treatment. [file Table_5.DOCX]

| Table 5: Proteins affected by risperidone treatment | | | | |
| --- | --- | --- | --- | --- |
| *Accession* | *Gene* | *Anova (p)* | *Log2 FC* | *Protein* |
| Q92878 | RAD50 | 0,00079 | -11,7158 | DNA repair protein RAD50 |
| Q9NX24 | NHP2 | 8,49E-05 | -9,34401 | H/ACA ribonucleoprotein complex subunit 2 |
| P54727 | RAD23B | 0,001218 | -7,05572 | UV excision repair protein RAD23 homolog B |
| P61204 | ARF3 | 0,004313 | -6,219 | ADP-ribosylation factor 3 |
| O75844 | ZMPSTE24 | 0,008974 | -5,90818 | CAAX prenyl protease 1 homolog |
| P52788 | SMS | 0,037007 | -5,88906 | Spermine synthase |
| Q9H6Z4 | RANBP3 | 0,000268 | -5,80556 | Ran-binding protein 3 |
| Q9BYD6 | MRPL1 | 0,00424 | -5,70158 | 39S ribosomal protein L1_ mitochondrial |
| P61619 | SEC61A1 | 0,025632 | -5,41593 | Protein transport protein Sec61 subunit alpha isoform 1 |
| Q9C005 | DPY30 | 0,003887 | -5,23031 | Protein dpy-30 homolog |
| P11488 | GNAT1 | 0,000699 | -5,11909 | Guanine nucleotide-binding protein G(t) subunit alpha-1 |
| P37268 | FDFT1 | 0,000657 | -4,78825 | Squalene synthase |
| Q8N684 | CPSF7 | 0,013616 | -4,67307 | Cleavage and polyadenylation specificity factor subunit 7 |
| Q7L2E3 | DHX30 | 0,00954 | -3,99573 | Putative ATP-dependent RNA helicase DHX30 |
| Q9P0L0 | VAPA | 0,000747 | -3,904 | Vesicle-associated membrane protein-associated protein A |
| Q99848 | EBNA1BP2 | 0,04308 | -3,87269 | Probable rRNA-processing protein EBP2 |
| Q9H4L4 | SENP3 | 0,015529 | -3,75398 | Sentrin-specific protease 3 |
| Q92979 | EMG1 | 0,006564 | -3,73788 | Ribosomal RNA small subunit methyltransferase NEP1 |
| Q9Y2Q3 | GSTK1 | 0,037022 | -3,5717 | Glutathione S-transferase kappa 1 |
| P26368 | U2AF2 | 0,004871 | -3,50381 | Splicing factor U2AF 65 kDa subunit |
| Q9BSJ8 | ESYT1 | 0,009553 | -3,47147 | Extended synaptotagmin-1 |
| Q6DKI1 | RPL7L1 | 0,019562 | -3,36167 | 60S ribosomal protein L7-like 1 |
| Q92930 | RAB8B | 0,007052 | -3,30281 | Ras-related protein Rab-8B |
| O14776 | TCERG1 | 0,011498 | -3,29103 | Transcription elongation regulator 1 |
| Q5EB52 | MEST | 0,017442 | -3,27129 | Mesoderm-specific transcript homolog protein |
| A1L0T0 | ILVBL | 0,034499 | -3,22103 | Acetolactate synthase-like protein |
| Q9NXF1 | TEX10 | 0,048823 | -3,19796 | Testis-expressed protein 10 |
| Q9NW64 | RBM22 | 0,004899 | -3,05338 | Pre-mRNA-splicing factor RBM22 |
| O60701 | UGDH | 0,046047 | -3,01033 | UDP-glucose 6-dehydrogenase |
| P30046 | DDT | 0,013187 | -3,00556 | D-dopachrome decarboxylase |
| P48729 | CSNK1A1 | 0,00458 | -2,98945 | Casein kinase I isoform alpha |
| O60264 | SMARCA5 | 0,006003 | -2,96891 | SWI/SNF-related matrix-associated actin-dependent regulator of chromatin subfamily A member 5 |
| Q6UXN9 | WDR82 | 0,0306 | -2,91521 | WD repeat-containing protein 82 |
| P35637 | FUS | 0,000494 | -2,85656 | RNA-binding protein FUS |
| P26358 | DNMT1 | 0,018039 | -2,82634 | DNA (cytosine-5)-methyltransferase 1 |
| Q9UQE7 | SMC3 | 0,008389 | -2,76863 | Structural maintenance of chromosomes protein 3 |
| P33993 | MCM7 | 0,001079 | -2,73282 | DNA replication licensing factor MCM7 |
| P28331 | NDUFS1 | 0,004927 | -2,69422 | NADH-ubiquinone oxidoreductase 75 kDa subunit_ mitochondrial |
| Q15428 | SF3A2 | 0,010973 | -2,67409 | Splicing factor 3A subunit 2 |
| Q9Y2W1 | THRAP3 | 0,011115 | -2,67312 | Thyroid hormone receptor-associated protein 3 |
| Q9ULC4 | MCTS1 | 0,011396 | -2,66943 | Malignant T-cell-amplified sequence 1 |
| P59998 | ARPC4 | 0,003979 | -2,59203 | Actin-related protein 2/3 complex subunit 4 |
| B5ME19 | EIF3CL | 0,026584 | -2,57324 | Eukaryotic translation initiation factor 3 subunit C-like protein |
| Q07021 | C1QBP | 0,016399 | -2,56948 | Complement component 1 Q subcomponent-binding protein_ mitochondrial |
| Q8IY81 | FTSJ3 | 0,025891 | -2,50908 | pre-rRNA processing protein FTSJ3 |
| P46779 | RPL28 | 0,047309 | -2,49086 | 60S ribosomal protein L28 |
| O75821 | EIF3G | 0,001315 | -2,48614 | Eukaryotic translation initiation factor 3 subunit G |
| P04908 | HIST1H2AB | 0,004888 | -2,46809 | Histone H2A type 1-B/E |
| Q9NYF8 | BCLAF1 | 0,006967 | -2,43559 | Bcl-2-associated transcription factor 1 |
| Q5JTH9 | RRP12 | 0,017107 | -2,3813 | RRP12-like protein |
| Q99459 | CDC5L | 0,005506 | -2,33707 | Cell division cycle 5-like protein |
| P11233 | RALA | 0,032144 | -2,32443 | Ras-related protein Ral-A |
| P51610 | HCFC1 | 0,036417 | -2,30271 | Host cell factor 1 |
| Q9UKA9 | PTBP2 | 0,023683 | -2,2721 | Polypyrimidine tract-binding protein 2 |
| Q14151 | SAFB2 | 0,023809 | -2,25542 | Scaffold attachment factor B2 |
| Q8NBU5 | ATAD1 | 0,002782 | -2,25135 | ATPase family AAA domain-containing protein 1 |
| P09234 | SNRPC | 0,010691 | -2,23578 | U1 small nuclear ribonucleoprotein C |
| Q86YP4 | GATAD2A | 0,008046 | -2,22599 | Transcriptional repressor p66-alpha |
| Q8IY67 | RAVER1 | 0,047837 | -2,209 | Ribonucleoprotein PTB-binding 1 |
| Q8NHY3 | GAS2L2 | 0,009335 | -2,20473 | GAS2-like protein 2 |
| P31949 | S100A11 | 0,012856 | -2,19448 | Protein S100-A11 |
| Q14103 | HNRNPD | 0,00168 | -2,15808 | Heterogeneous nuclear ribonucleoprotein D0 |
| Q14683 | SMC1A | 0,010684 | -2,15675 | Structural maintenance of chromosomes protein 1A |
| O43684 | BUB3 | 0,007179 | -2,13262 | Mitotic checkpoint protein BUB3 |
| Q5BKZ1 | ZNF326 | 0,010144 | -2,11663 | DBIRD complex subunit ZNF326 |
| P23193 | TCEA1 | 0,034038 | -2,05835 | Transcription elongation factor A protein 1 |
| Q07666 | KHDRBS1 | 0,030449 | -2,05676 | KH domain-containing_ RNA-binding_ signal transduction-associated protein 1 |
| Q9UBS4 | DNAJB11 | 0,00901 | -2,04004 | DnaJ homolog subfamily B member 11 |
| Q9NS69 | TOMM22 | 0,042063 | -2,03372 | Mitochondrial import receptor subunit TOM22 homolog |
| Q14141 | SEPT6 | 0,025366 | -2,00119 | Septin-6 |
| O43852 | CALU | 0,001569 | -1,99781 | Calumenin |
| P63173 | RPL38 | 0,041731 | -1,99683 | 60S ribosomal protein L38 |
| O60832 | DKC1 | 0,009501 | -1,98602 | H/ACA ribonucleoprotein complex subunit 4 |
| P62847 | RPS24 | 0,019559 | -1,98415 | 40S ribosomal protein S24 |
| P17480 | UBTF | 0,02053 | -1,98169 | Nucleolar transcription factor 1 |
| P55795 | HNRNPH2 | 0,023214 | -1,93132 | Heterogeneous nuclear ribonucleoprotein H2 |
| Q9UL46 | PSME2 | 0,040462 | -1,90751 | Proteasome activator complex subunit 2 |
| P17844 | DDX5 | 0,02715 | -1,88877 | Probable ATP-dependent RNA helicase DDX5 |
| P31943 | HNRNPH1 | 0,020261 | -1,85173 | Heterogeneous nuclear ribonucleoprotein H |
| P82979 | SARNP | 0,031541 | -1,77945 | SAP domain-containing ribonucleoprotein |
| Q49A26 | GLYR1 | 0,02159 | -1,77888 | Putative oxidoreductase GLYR1 |
| Q15370 | ELOB | 0,039991 | -1,76563 | Elongin-B |
| P41250 | GARS | 0,005028 | -1,74181 | Glycine--tRNA ligase |
| P07197 | NEFM | 0,003457 | -1,73528 | Neurofilament medium polypeptide |
| Q16629 | SRSF7 | 0,006212 | -1,7026 | Serine/arginine-rich splicing factor 7 |
| Q8NBS9 | TXNDC5 | 0,01728 | -1,69915 | Thioredoxin domain-containing protein 5 |
| Q15041 | ARL6IP1 | 0,006388 | -1,69019 | ADP-ribosylation factor-like protein 6-interacting protein 1 |
| Q15233 | NONO | 0,020284 | -1,6868 | Non-POU domain-containing octamer-binding protein |
| Q9UDY4 | DNAJB4 | 0,007967 | -1,66733 | DnaJ homolog subfamily B member 4 |
| Q9Y4L1 | HYOU1 | 0,035083 | -1,66417 | Hypoxia up-regulated protein 1 |
| P55265 | ADAR | 0,016832 | -1,63093 | Double-stranded RNA-specific adenosine deaminase |
| Q9NQ29 | LUC7L | 0,012279 | -1,62595 | Putative RNA-binding protein Luc7-like 1 |
| Q9H7B2 | RPF2 | 0,019548 | -1,62577 | Ribosome production factor 2 homolog |
| Q14690 | PDCD11 | 0,026891 | -1,6226 | Protein RRP5 homolog |
| Q9BRJ6 | C7orf50 | 0,014741 | -1,60571 | Uncharacterized protein C7orf50 |
| P61970 | NUTF2 | 0,021834 | -1,5945 | Nuclear transport factor 2 |
| Q9HB71 | CACYBP | 0,001435 | -1,57761 | Calcyclin-binding protein |
| P57721 | PCBP3 | 0,048914 | -1,57178 | Poly(rC)-binding protein 3 |
| P14778 | IL1R1 | 0,044228 | -1,56152 | Interleukin-1 receptor type 1 |
| O15523 | DDX3Y | 0,015637 | -1,55347 | ATP-dependent RNA helicase DDX3Y |
| O14979 | HNRNPDL | 0,034675 | -1,552 | Heterogeneous nuclear ribonucleoprotein D-like |
| O00170 | AIP | 0,029451 | -1,5499 | AH receptor-interacting protein |
| P05387 | RPLP2 | 0,016285 | -1,54869 | 60S acidic ribosomal protein P2 |
| P12004 | PCNA | 0,004059 | -1,53065 | Proliferating cell nuclear antigen |
| P09651 | HNRNPA1 | 0,029919 | -1,53063 | Heterogeneous nuclear ribonucleoprotein A1 |
| P05141 | SLC25A5 | 0,013244 | -1,52578 | ADP/ATP translocase 2 |
| P19338 | NCL | 0,000193 | -1,52243 | Nucleolin |
| Q9P258 | RCC2 | 0,031561 | -1,51869 | Protein RCC2 |
| P08138 | NGFR | 0,028596 | -1,5122 | Tumor necrosis factor receptor superfamily member 16 |
| Q15366 | PCBP2 | 0,040454 | -1,48655 | Poly(rC)-binding protein 2 |
| Q00839 | HNRNPU | 0,043054 | -1,47855 | Heterogeneous nuclear ribonucleoprotein U |
| Q96A08 | HIST1H2BA | 0,034628 | -1,46574 | Histone H2B type 1-A |
| P04264 | KRT1 | 0,029386 | -1,46539 | Keratin_ type II cytoskeletal 1 |
| P01023 | A2M | 0,020865 | -1,39549 | Alpha-2-macroglobulin |
| Q15046 | KARS | 0,027107 | -1,36987 | Lysine--tRNA ligase |
| O43809 | NUDT21 | 0,03737 | -1,36817 | Cleavage and polyadenylation specificity factor subunit 5 |
| P23284 | PPIB | 0,00405 | -1,35987 | Peptidyl-prolyl cis-trans isomerase B |
| Q9UDY2 | TJP2 | 3,72E-05 | -1,34534 | Tight junction protein ZO-2 |
| O15511 | ARPC5 | 0,038554 | -1,33978 | Actin-related protein 2/3 complex subunit 5 |
| Q9ULW0 | TPX2 | 0,02846 | -1,33625 | Targeting protein for Xklp2 |
| Q0IIM8 | TBC1D8B | 0,007476 | -1,30647 | TBC1 domain family member 8B |
| P13861 | PRKAR2A | 0,000809 | -1,30429 | cAMP-dependent protein kinase type II-alpha regulatory subunit |
| P43243 | MATR3 | 0,006615 | -1,29672 | Matrin-3 |
| Q8IZP2 | ST13P4 | 0,014443 | -1,24729 | Putative protein FAM10A4 |
| O75385 | ULK1 | 0,006301 | -1,22589 | Serine/threonine-protein kinase ULK1 |
| O14980 | XPO1 | 0,039642 | -1,22148 | Exportin-1 |
| P62861 | FAU | 0,037677 | -1,20706 | 40S ribosomal protein S30 |
| O76021 | RSL1D1 | 0,022297 | -1,20241 | Ribosomal L1 domain-containing protein 1 |
| Q92841 | DDX17 | 0,000779 | -1,17652 | Probable ATP-dependent RNA helicase DDX17 |
| P45973 | CBX5 | 0,03215 | -1,17369 | Chromobox protein homolog 5 |
| Q14240 | EIF4A2 | 0,024144 | -1,16451 | Eukaryotic initiation factor 4A-II |
| P46060 | RANGAP1 | 0,014792 | -1,13876 | Ran GTPase-activating protein 1 |
| P40926 | MDH2 | 0,049654 | -1,12361 | Malate dehydrogenase_ mitochondrial |
| P09661 | SNRPA1 | 0,020105 | -1,12108 | U2 small nuclear ribonucleoprotein A' |
| P16070 | CD44 | 0,048587 | -1,10883 | CD44 antigen |
| Q9GZQ8 | MAP1LC3B | 0,032946 | -1,09678 | Microtubule-associated proteins 1A/1B light chain 3B |
| P34931 | HSPA1L | 0,023361 | -1,07758 | Heat shock 70 kDa protein 1-like |
| P52272 | HNRNPM | 0,042548 | -1,02655 | Heterogeneous nuclear ribonucleoprotein M |
| Q96PV4 | PNMA5 | 0,019111 | -1,0249 | Paraneoplastic antigen-like protein 5 |
| Q86SE5 | RALYL | 0,016582 | -0,98832 | RNA-binding Raly-like protein |
| P49207 | RPL34 | 0,018933 | -0,97529 | 60S ribosomal protein L34 |
| Q9UQ80 | PA2G4 | 0,026811 | -0,96892 | Proliferation-associated protein 2G4 |
| Q13151 | HNRNPA0 | 0,004738 | -0,95521 | Heterogeneous nuclear ribonucleoprotein A0 |
| P23921 | RRM1 | 0,030943 | -0,94771 | Ribonucleoside-diphosphate reductase large subunit |
| P22314 | UBA1 | 0,033825 | -0,91221 | Ubiquitin-like modifier-activating enzyme 1 |
| P23246 | SFPQ | 0,039945 | -0,90419 | Splicing factor_ proline- and glutamine-rich |
| P49458 | SRP9 | 0,035743 | -0,89918 | Signal recognition particle 9 kDa protein |
| P83731 | RPL24 | 0,049773 | -0,87895 | 60S ribosomal protein L24 |
| P21333 | FLNA | 0,039758 | -0,87891 | Filamin-A |
| Q9Y4Z0 | LSM4 | 0,020154 | -0,87799 | U6 snRNA-associated Sm-like protein LSm4 |
| P35659 | DEK | 0,02423 | -0,87602 | Protein DEK |
| P32969 | RPL9 | 0,030195 | -0,82644 | 60S ribosomal protein L9 |
| P53621 | COPA | 0,020506 | -0,77882 | Coatomer subunit alpha |
| Q9UQ35 | SRRM2 | 0,008351 | -0,75485 | Serine/arginine repetitive matrix protein 2 |
| P37837 | TALDO1 | 0,034207 | -0,71401 | Transaldolase |
| P07339 | CTSD | 0,025648 | -0,6802 | Cathepsin D |
| Q01130 | SRSF2 | 0,007119 | -0,65216 | Serine/arginine-rich splicing factor 2 |
| Q9BRL6 | SRSF8 | 0,007119 | -0,65216 | Serine/arginine-rich splicing factor 8 |
| Q9Y230 | RUVBL2 | 0,011549 | 0,842904 | RuvB-like 2 |
| P12268 | IMPDH2 | 0,034642 | 0,870704 | Inosine-5'-monophosphate dehydrogenase 2 |
| O00425 | IGF2BP3 | 0,047216 | 0,892895 | Insulin-like growth factor 2 mRNA-binding protein 3 |
| P33992 | MCM5 | 0,019443 | 0,899644 | DNA replication licensing factor MCM5 |
| P22234 | PAICS | 0,009679 | 0,946094 | Multifunctional protein ADE2 |
| Q96AC1 | FERMT2 | 0,048051 | 1,105142 | Fermitin family homolog 2 |
| Q9Y262 | EIF3L | 0,02927 | 1,155677 | Eukaryotic translation initiation factor 3 subunit L |
| Q13242 | SRSF9 | 0,004151 | 1,180974 | Serine/arginine-rich splicing factor 9 |
| P49327 | FASN | 0,047714 | 1,244966 | Fatty acid synthase |
| P08621 | SNRNP70 | 0,006008 | 1,296447 | U1 small nuclear ribonucleoprotein 70 kDa |
| P51148 | RAB5C | 0,048 | 1,322594 | Ras-related protein Rab-5C |
| Q13283 | G3BP1 | 0,03175 | 1,405822 | Ras GTPase-activating protein-binding protein 1 |
| Q9NZI8 | IGF2BP1 | 0,027992 | 1,625785 | Insulin-like growth factor 2 mRNA-binding protein 1 |
| P82932 | MRPS6 | 0,031131 | 1,674477 | 28S ribosomal protein S6_ mitochondrial |
| Q15459 | SF3A1 | 0,017877 | 1,722919 | Splicing factor 3A subunit 1 |
| P18621 | RPL17 | 0,03033 | 2,01346 | 60S ribosomal protein L17 |
| P26583 | HMGB2 | 0,028115 | 2,022939 | High mobility group protein B2 |
| Q15056 | EIF4H | 0,022851 | 2,027248 | Eukaryotic translation initiation factor 4H |
| Q71UM5 | RPS27L | 0,026062 | 2,129703 | 40S ribosomal protein S27-like |
| Q9Y3A5 | SBDS | 0,026118 | 2,268257 | Ribosome maturation protein SBDS |
| O75964 | ATP5L | 0,044582 | 2,278426 | ATP synthase subunit g_ mitochondrial |
| P31040 | SDHA | 0,010153 | 2,330392 | Succinate dehydrogenase [ubiquinone] flavoprotein subunit_ mitochondrial |
| Q86UQ4 | ABCA13 | 0,02365 | 2,456327 | ATP-binding cassette sub-family A member 13 |
| P22570 | FDXR | 0,037822 | 2,473715 | NADPH:adrenodoxin oxidoreductase_ mitochondrial |
| P16591 | FER | 0,003173 | 2,530129 | Tyrosine-protein kinase Fer |
| Q92499 | DDX1 | 0,035018 | 2,65584 | ATP-dependent RNA helicase DDX1 |
| Q9UK76 | JPT1 | 0,017537 | 2,707331 | Jupiter microtubule associated homolog 1 |
| Q9NQ50 | MRPL40 | 0,013956 | 2,751963 | 39S ribosomal protein L40_ mitochondrial |
| Q9GZT3 | SLIRP | 0,009257 | 2,852685 | SRA stem-loop-interacting RNA-binding protein_ mitochondrial |
| Q9HCK8 | CHD8 | 0,029397 | 2,995818 | Chromodomain-helicase-DNA-binding protein 8 |
| Q5JQF8 | PABPC1L2A | 0,015638 | 3,280386 | Polyadenylate-binding protein 1-like 2 |
| Q13554 | CAMK2B | 0,002166 | 3,683994 | Calcium/calmodulin-dependent protein kinase type II subunit beta |
| P54886 | ALDH18A1 | 0,007884 | 4,147364 | Delta-1-pyrroline-5-carboxylate synthase |
| Q3L8U1 | CHD9 | 0,025419 | 4,14772 | Chromodomain-helicase-DNA-binding protein 9 |
| Q96CW1 | AP2M1 | 0,002183 | 4,194245 | AP-2 complex subunit mu |
| Q96FW1 | OTUB1 | 0,024469 | 4,299616 | Ubiquitin thioesterase OTUB1 |
| Q9H2D6 | TRIOBP | 0,020193 | 4,587974 | TRIO and F-actin-binding protein |
