# Supplementary material for: Biochemical Pathways Triggered by Antipsychotics in Human Oligodendrocytes: Potential of Discovering New Treatment Targets
Source: Front Pharmacol. 2019 Mar 5;10:186. doi: 10.3389/fphar.2019.00186 (PMC6411851; doi:10.3389/fphar.2019.00186)
Supplement: Table S6 — Ingenuity canonical pathway analysis for oligodendrocyte treated with second generation antipsychotics. [file Table_6.DOCX]

| **Table 6 - Ingenuity canonical pathway analysis for oligodendrocyte treated with second generation antipsychotics** | | | | | | | |
| --- | --- | --- | --- | --- | --- | --- | --- |
| Treatment | Ingenuity Canonical Pathways | p-value | Ratio | Treatment | Ingenuity Canonical Pathways | p-value | Ratio |
| Risperidone | EIF2 Signaling | 1,12202E-09 | 15/221 (0,0679) | Quetiapine | Myc Mediated Apoptosis Signaling | 0,001479108 | 2/70 (0,0286) |
| Risperidone | mTOR Signaling | 0,001995262 | 7/201 (0,0348) | Quetiapine | Granzyme B Signaling | 0,013182567 | 1/16 (0,0625) |
| Risperidone | Regulation of eIF4 and p70S6K Signaling | 0,002630268 | 6/157 (0,0382) | Quetiapine | Parkinson's Signaling | 0,013182567 | 1/16 (0,0625) |
| Risperidone | Remodeling of Epithelial Adherens Junctions | 0,003162278 | 4/69 (0,058) | Quetiapine | Systemic Lupus Erythematosus Signaling | 0,014454398 | 2/227 ()0,00881 |
| Risperidone | Purine Nucleotides De Novo Biosynthesis II | 0,003981072 | 2/11 (0,182) | Quetiapine | Huntington's Disease Signaling | 0,017378008 | 2/250 (0,008) |
| Risperidone | Role of p14/p19ARF in Tumor Suppression | 0,006309573 | 3/43 (0,0698) | Quetiapine | Tumoricidal Function of Hepatic Natural Killer Cells | 0,019498446 | 1/24 (0,0417) |
| Risperidone | Telomere Extension by Telomerase | 0,007413102 | 2/15 (0,133) | Quetiapine | Cytotoxic T Lymphocyte-mediated Apoptosis of Target Cells | 0,025703958 | 1/32 (0,0312) |
| Risperidone | Aryl Hydrocarbon Receptor Signaling | 0,008128305 | 5/141 (0,0355) | Quetiapine | TWEAK Signaling | 0,028183829 | 1/35 (0,0286) |
| Risperidone | RAN Signaling | 0,009549926 | 2/17 (0,118) | Quetiapine | Nucleotide Excision Repair Pathway | 0,028183829 | 1/35 (0,0286) |
| Risperidone | ATM Signaling | 0,010715193 | 4/98 (0,0408) | Quetiapine | Role of PKR in Interferon Induction and Antiviral Response | 0,033113112 | 1/41 (0,0244) |
| Risperidone | Cell Cycle Control of Chromosomal Replication | 0,012882496 | 3/56 (0,0536) | Quetiapine | TNFR1 Signaling | 0,039810717 | 1/50 (0,02) |
| Risperidone | Sirtuin Signaling Pathway | 0,014791084 | 7/292 (0,024) | Quetiapine | Assembly of RNA Polymerase II Complex | 0,039810717 | 1/50 (0,02) |
| Risperidone | Tight Junction Signaling | 0,015848932 | 5/167 (0,0299) | Quetiapine | Cell Cycle: G2/M DNA Damage Checkpoint Regulation | 0,039810717 | 1/50 (0,02) |
| Risperidone | autophagy | 0,016982437 | 3/62 (0,0484) | Quetiapine | Docosahexaenoic Acid (DHA) Signaling | 0,041686938 | 1/52 (0,0192) |
| Risperidone | Spliceosomal Cycle | 0,017378008 | 1/2 (0,5) | Quetiapine | CD27 Signaling in Lymphocytes | 0,042657952 | 1/53 (0,0189) |
| Risperidone | UDP-D-xylose and UDP-D-glucuronate Biosynthesis | 0,017378008 | 1/2 (0,5) | Quetiapine | Nur77 Signaling in T Lymphocytes | 0,047863009 | 1/59 (0,0169) |
| Risperidone | Epoxysqualene Biosynthesis | 0,017378008 | 1/2 (0,5) | Quetiapine | Induction of Apoptosis by HIV1 | 0,048977882 | 1/61 (0,0164) |
| Risperidone | Spermine Biosynthesis | 0,017378008 | 1/2 (0,5) |  |  |  |  |
| Risperidone | Palmitate Biosynthesis I (Animals) | 0,017378008 | 1/2 (0,5) |  |  |  |  |
| Risperidone | Fatty Acid Biosynthesis Initiation II | 0,017378008 | 1/2 (0,5) |  |  |  |  |
| Risperidone | TCA Cycle II (Eukaryotic) | 0,018620871 | 2/24 (0,0833) |  |  |  |  |
| Risperidone | LXR/RXR Activation | 0,021877616 | 4/121 (0,0331) |  |  |  |  |
| Risperidone | Caveolar-mediated Endocytosis Signaling | 0,024547089 | 3/71 (0,0423) |  |  |  |  |
| Risperidone | Inosine-5'-phosphate Biosynthesis II | 0,02630268 | 1/3 (0,333) |  |  |  |  |
| Risperidone | Thyroid Hormone Biosynthesis | 0,02630268 | 1/3 (0,333) |  |  |  |  |
| Risperidone | Oxidized GTP and dGTP Detoxification | 0,02630268 | 1/3 (0,333) |  |  |  |  |
| Risperidone | Protein Ubiquitination Pathway | 0,029512092 | 6/265 (0,0226) |  |  |  |  |
| Risperidone | Proline Biosynthesis I | 0,034673685 | 1/4 (0,25) |  |  |  |  |
| Risperidone | Eumelanin Biosynthesis | 0,042657952 | 1/5 (0,2) |  |  |  |  |
| Risperidone | tRNA Charging | 0,045708819 | 2/39 (0,0513) |  |  |  |  |
| Risperidone | LPS/IL-1 Mediated Inhibition of RXR Function | 0,045708819 | 5/222 (0,0225) |  |  |  |  |
| Risperidone | IL-1 Signaling | 0,046773514 | 3/92 (0,0326) |  |  |  |  |
